# Maternal vaccination against RSV can substantially reduce childhood mortality in low-income and middle-income countries: A mathematical modeling study

**DOI:** 10.1016/j.jvacx.2023.100379

**Published:** 2023-09-01

**Authors:** Joukje E. Willemsen, José A.M. Borghans, Louis J. Bont, Julia Drylewicz

**Affiliations:** a Centre for Translational Immunology, University Medical Centre Utrecht, Utrecht, The Netherlands; b Division of Infectious Diseases, Department of Pediatrics, University Medical Centre Utrecht, Utrecht, The Netherlands

**Keywords:** Maternal vaccination, RSV, Vaccine efficacy, Mathematical model, Predictive modelling, Infant mortality, Vaccine model

## Abstract

**Background:**

Respiratory syncytial virus (RSV) is a leading cause of childhood mortality in infants below 6 months of age. In low-income and middle-income countries (LMICs), the public health burden is substantial and resources are limited. It is critical to inform decision makers about effectiveness of new interventions.

**Methods:**

We developed a mathematical model where individual RSV subtype A (RSV-A) and B (RSV-B) maternally derived neutralizing titers were predicted at time of birth after maternal vaccination with the RSV prefusion F protein-based vaccine. We estimated the subsequent duration of vaccine-induced immunity and compared this to the age at time of death distribution in the RSV GOLD Mortality Database to predict the potential impact of maternal vaccination on RSV-related childhood mortality. We used country-specific timing of antenatal care visits distributions and mortality estimates to make country-specific predictions for number of cases averted.

**Findings:**

The model predicts that on average a neonate born at 40 weeks gestational age will be protected between 6 and 7 months from RSV-A and approximately 5 months from RSV-B related mortality. We estimated the potential impact of RSV-related mortality for in-hospital and out-of-hospital cases in LMICs and predicted that in 51 GAVI-eligible countries maternal vaccination could avert between 55% and 63% of the RSV-related in-hospital mortality cases below 6 months of age.

**Interpretation:**

We show that maternal vaccination could substantially decrease RSV-A and RSV-B related in-hospital and out-of-hospital mortality in LMICs in the first 6 months of life.

## Highlights

• Maternal vaccination could substantially decrease RSV related mortality in LMICs in the first 6 months of life.
• The duration of vaccine-induced immunity depends strongly on gestational age at time of birth.
• For the RSVpreF vaccine the optimal timing of vaccination would be early in pregnancy.
• After reparameterization, our mathematical model for maternal vaccine-induced antibody dynamics can be applied to other vaccines that can be recommended during pregnancy.

## Introduction

Respiratory syncytial virus (RSV) is a leading cause of childhood morbidity and mortality in infants below 5 years of age [1]. RSV-associated acute lower respiratory infections are responsible for worldwide 3.6 million hospital admissions and 26,300 in-hospital deaths annually. The vast majority (97%) of these RSV-attributable deaths occur in low-income and middle-income countries (LMICs), and almost half of these RSV-attributable deaths occur in the first 6 months of life [1]. Furthermore, it is estimated that for every in-hospital RSV-attributable death across all age groups, there are three out-of-hospital RSV-attributable deaths [1].

Multiple RSV interventions are currently in development [2]. Active vaccination will not protect the youngest infants, among whom the burden is the highest, since it will likely require several doses to elicit a robust vaccine-derived immune response [3]. Passive vaccination, via administration of monoclonal antibodies or maternal vaccination will provide only temporary protection [4]. Maternal immunization strategies aim to protect very young infants by transferring antibodies across the placenta from mother to fetus [5]. Maternal vaccines are in late stage of clinical development, and are anticipated to become available in the coming years [6].

In LMICs, the public health burden is substantial and resources are limited. Thus, it is critical that governments and large funding organizations are informed about effectiveness of new interventions so that interventions that have the highest impact and best value for money can be prioritized [3]. Seasonal dosing administration approaches in places with clear seasonality are considered to enhance the cost-effectiveness of maternal vaccination [4]. To optimize the RSV vaccination strategy, it is essential to know the duration of protection by maternal vaccination.

The maternal vaccine known as RSVpreF, marketed under the brand name Abrysvo, has recently received approval from the European Medicines Agency (EMA) and could soon be available [7]. The corresponding phase 3 trial was conducted in 18 countries and included healthy pregnant women, 49 years of age or younger, at 24 through 36 weeks’ gestation on the day of planned injection [6]. While a vaccine’s efficacy is measured in a controlled clinical trial before approval, clinical trials alone will provide insufficient information to extrapolate vaccine efficacy to different populations due to the diversity in epidemiological settings in which preventive strategies could be applied [3]. Furthermore, the efficacy of maternal vaccines in specific risk groups, such as prematurely born infants, remain understudied in clinical trials due to the lack of statistical power [8]. Mathematical models represent a solution for studying the potential impact of different preventive strategies, depending on various vaccine characteristics and setting-specific health system factors without greatly increasing the size of trials [3]. Vaccine effectiveness estimates feed into cost-effectiveness analyses, used by policy makers, to prioritize interventions that have the highest impact and best value for money. Mathematical modeling could contribute to predict vaccine cost-effectiveness and inform decision making for governments and large funding organizations such as Gavi, the vaccine alliance [5].

Gavi considered some of the RSV interventions under development, including both maternal vaccination and monoclonal antibodies in its 2018 vaccine investment strategy for the 2021–2025 funding period. Health impact defined by the number of cases and deaths averted was used as one of the main criteria for prioritizing interventions [9]. There is however a paucity of data in LMICs on hospitalization and mortality in the current RSV modeling literature, specifically lacking geographical coverage [3]. In addition, out-of-hospital mortality has not been fully evaluated in the literature on RSV in LMICs due to a lack of data, despite the high burden of RSV morbidity in the population.

The present study predicts country-specific impact of maternal vaccination in LMICs on both in-hospital and out-of-hospital mortality. We show that our mathematical model provides a useful tool to make subgroup-specific predictions for future vaccine impact and to explore the impact of timing of vaccination and gestational age at time of birth on vaccine efficacy.

## Material and methods

### Mathematical model

As described previously [10], individual antibody titers of mother and infant pairs were modelled over time. We have made a few improvements compared to the model previously published. We decided to parameterize our model on the RSV prefusion F protein-based (RSVpreF) maternal vaccine [11], as this vaccine might already be registered in 2023 [12]. As immunogenity data for RSVpreF is reported in RSV subtype A (RSV-A) and B (RSV-B) neutralizing titers, we parameterized our model on RSV-A and RSV-B neutralizing antibodies. Furthermore, we assumed a peak in neutralizing activity in the mothers’ serum 14 days post vaccination [13], [14], [15], in contrast with the assumed peak after 21 days in [10]. Lastly, in contrast to [10], we added maternal antibody decline during pregnancy in the model to match the phase 1/2 RSV RSVpreF trial data that is currently under development [16], [11]. The decline of maternal antibody levels after vaccination has also been observed in the mothers in the phase 3 Novavax maternal vaccine trial [17].

We hence assumed that maternal RSV-A and RSV-B neutralizing antibody titers increase exponentially from 7 days after vaccination, until they reach a peak at 14 days. After 14 days, maternal antibody levels decline exponentially. The maternal antibody titers are described in Equation (1). Where t is the number of days since vaccination, $a_{m_{0}}$ is the maternal 50% neutralizing antibody titer (geometric mean titer level needed to prevent 50% of infections) before vaccination and f the fold increase in neutralizing antibody titers 14 days post vaccination.

(1) $$a_{m}\left(t\right)=\;\left\{\begin{array}{ll} a_{m_{0}} & if\;t<7\\ a_{m_{0}}*\;e^{\left(t-7\right)\log\left(f\right)/7} & if\;t\;⩽7\;⩽14\\ a_{m_{0}}*\;f*\;{\frac{1}{2}}^{\left(t-14\right)/t_{m1/2}} & if\;t<14 \end{array}\right)$$

The foetus-to-mother antibody transfer function was modelled as described previously [10], such that the neonatal antibody titers ($a_{n}$) at gestational age at time of birth in days ($t_{b}$) depend on both the antibody transfer function $r\left(t_{b}\right)$ and maternal antibody titers, $a_{m}\left(t\right)$, as follows:

(2) $$a_{n}\left(t_{b},\;t\right)=r\left(t_{b}\right)*a_{m}\left(t\right)$$

Using the individual antibody titers of mother-fetus pairs during pregnancy, we predicted the number of days protected (which we refer to as duration of vaccine-induced immunity) from RSV-related death ($T_{\mathrm{prot}}$) as described in Equation (3).

Where $t_{N_{\frac{1}{2}}}$ is the half-life in neonates in days and *A_prot_* is the protection threshold in 50% neutralizing antibody geometric mean titer.

(3) $$T_{\mathrm{prot}}\left(t_{b},\;t\right)=\frac{t_{N_{\frac{1}{2}}}*ln\left(\frac{A_{\mathrm{prot}}}{a_{n}\left(t_{b},\;t\right)}\right)}{-ln(2)}$$

Parameter values were fixed according to the published phase 1/2 RSVpreF vaccine trial [11] and are reported in Table 1. We estimated the fold-increase at 14 days post vaccination and the half-life of vaccine induced antibodies in the mother based on the reported RSV-A and RSV-B neutralizing geometric mean fold rises post vaccination in [11] (See supplemental materials S2 for more details).

**Table 1:** Parameter values of the model.

Parameter	Abbreviation	RSV-A	RSV-B	Source
Prevaccination antibody titers (50% neutralizing GMT)	$a_{m_{0}}$	1434	1366	[11]
Fold increase 14 days post vaccination*	f	12.6	14.7	[11]
Half-life of maternal antibodies* (days)	$t_{M_{\frac{1}{2}}}$	274.5	229.5	[11]
Half-life of neonatal antibodies (days)	$t_{N_{\frac{1}{2}}}$	45.4	39.3	[19]
Protection threshold (50% neutralizing GMT)	$A_{\mathrm{prot}}$	903	2069	[11]

GMT = geometric mean titer, * = estimated based on available data in source.

Data on the decay of passively transferred antibodies are not yet available for the RSVpreF vaccine [18]. Therefore, we used data on the half-life in neonates of passively transferred RSV-A and RSV-B neutralizing antibodies in the phase 2 RSV F vaccine trial [19], which is in close agreement with reported values in previous clinical studies [10]. We assumed that RSV neutralizing titers corresponding to palivizumab serum titers, equal or above 100 µg/ml could avert (or at least postpone) RSV-related mortality [11].

### RSV GOLD database

We made use of the RSV Global Online Mortality Database [20] for predicting the potential impact of maternal vaccination on RSV-related childhood mortality. In short, RSV GOLD is an ongoing global online registry for infants under the age of 5 years who died with laboratory-confirmed RSV infection after January 1, 1995 [20]. Age at time of death is reported in days or in months by the collaborators. The variables collected in the RSV GOLD database and method of data collection have been published previously [20], [21], [22]. Two community studies who shared data with the RSV GOLD database only included children below 6 months of age [22]. To prevent selection bias in the age distribution, we included all in-hospital deaths and out-of-hospital deaths from infants in LMICs who died before 6 months of age in this study. This subset comprises 473 in-hospital deaths and 156 in-community deaths. Table S2 presents the clinical characteristics of the infants in these respective groups. Additionally, Fig. S4b displays world maps illustrating the distribution of cases from various countries. The data from this subset has been published previously [22]. We decided to simulate data distributions to create more representative data. We simulated 1000 age at time of death distributions for both out-of-hospital deaths and in-hospital deaths (See supplemental materials S1 for more details).

### Predicting vaccine efficacy

We define vaccine efficacy (*E*) as the fraction of the number of deaths (*N*) in the dataset that could have been averted, had the mothers been vaccinated. The simplest approach would be to compare the observed time of death (*Tdeath*) with the estimated duration of vaccine-induced immunity (Tprot) had the mother been vaccinated during pregnancy:

(4) $$E=\frac{1}{N}\;\sum_{i\;=1}^{N}\;f\left({\mathrm{Tprot}}_{i}\right)$$

Where

$$f\left({\mathrm{Tprot}}_{i}\right)\left\{\begin{array}{l} 0,\;\;\text{i}\text{f }{\mathrm{Tprot}}_{i}<{\mathrm{Tdeath}}_{i}\\ 1,\;\;\text{i}\text{f }{\mathrm{Tprot}}_{i}\geq {\mathrm{Tdeath}}_{i} \end{array}\right)$$

However, due to missing data on gestational age and potential bias in the reported age at time of death, we used simulated data distributions, to create more representative data instead of individual observations. To save computational time, we directly compare the estimated duration of vaccine-induced immunity with the simulated age at time of death distributions. We thereby estimate the probability that RSV-related mortality would have been averted ($p_{\mathrm{averted}}$) by maternal vaccination, depending on gestational age at time of vaccination ($t_{v}$) and gestational age at time of birth ($t_{b}$). The probability that *Tprot* ≥ *Tdeath* is estimated by taking the average area under the age at time of death curve, as follows:

(5) $$p_{\mathrm{averted}}\left(t_{v},t_{b}\right)=\frac{1}{N_{\mathrm{agedist}}}\sum_{j=1}^{N_{\mathrm{agedist}}}\int_{0}^{T_{\mathrm{prot}}}f_{j}\left(x\right)dx$$

where $N_{\mathrm{agedist}}$ is the number of simulated age distributions and *f _j_* the function for the age at time of death distribution. We weighted the probability estimates $p_{\mathrm{averted}}\left(t_{v},t_{b}\right)$ by the estimated probability mass function (Fig. S6b) for gestational age at time of birth as follows:

(6) $$p_{\mathrm{weigthed}}\left(t_{v}\right)=\frac{p_{\mathrm{averted}}\left(t_{v},t_{b}\right)*w\left(t_{b}\right)}{\sum w(t_{b})}$$

where $\frac{w(t_{b})}{\sum w(t_{b})}$ represents the estimated probability that $t_{b}$ occurs in the population. This yields the estimated vaccine impact in the population, in terms of mortality cases averted, given the assumed timing of vaccination.

To evaluate the effect of timing of maternal vaccination we considered an administration window for maternal vaccination between 24 and 36 weeks of gestation, as suggested in the current guidelines of the Phase 3 RSVpreF vaccine trial [23].

### Country-specific predictions on vaccine impact

Antenatal care (ANC) visits could be an easy access to administer vaccines that require strict gestational age windows of administration [24]. We hence argue that the country-specific distribution of time of ANC visits within the administration window (between 24 and 36 weeks) could serve as a proxy for the likelihood distribution for timing of maternal vaccination. Data on the projected proportion of ANC attenders was provided by Baral et al. [24]. Their paper includes a summary illustrating the variability of antenatal care (ANC) coverage estimates across different countries plot (Fig. 2 in [24]). They also provide proxy estimates pertaining to service availability and acceptance (S3 appendix in [24]).

To estimate the probability mass distribution for time of vaccination we summed the proportion of ANC attenders for visit 1, 2 and 3 together per week. The weekly total was divided by the summed total of the weekly proportions to create a probability mass distribution for time of vaccination ($g_{t_{v}}$).

$$g_{t_{v}}\left(t\right)=\;\frac{f_{V_{1}}\left(t\right)+\;f_{V_{2}}\left(t\right)+\;f_{V_{3}}\left(t\right)}{\sum_{24}^{36}\left(f_{V_{1}}\left(t\right)+\;f_{V_{2}}\left(t\right)+\;f_{V_{3}}\left(t\right)\right)}\;for\;24\;⩽\;t\;⩽36$$

Where $f_{V_{1}}\left(t\right)$, $f_{V_{2}}\left(t\right)$ and $f_{V_{3}}\left(t\right)$ are the probability mass functions of ANC visit 1, visit 2, and visit 3 attendance.

Assuming that all eligible women receive a vaccine, which we define as achieving full vaccine coverage, we estimate the vaccine efficacy per country ($E_{\mathrm{country}}$) by weighting the median percentage averted given time of vaccination by the probability mass distribution for each week within the administration window:

(7) $$E_{\mathrm{country}}=\sum_{24}^{36}P_{\mathrm{averted}}\left(t_{v}\right)*g_{t_{v}}\left(t_{v}\right)$$

For a more realistic vaccine efficacy estimate we multiplied $E_{\mathrm{country}}$ by the estimated service availability and acceptance proxy reported by Baral et al. [24].

Lastly, country-specific RSV-related mortality burden estimates reported by Li et al. [25] were multiplied by the predicted percentage averted given realistic vaccine coverage, to predict the number of mortality cases averted yearly per country. For this step we only included countries where both the service availability and acceptance proxy was reported [24] and where age-specific mortality estimates were available [25]. As [25] only estimated country-specific in-hospital mortality rates, we multiplied the mortality burden estimates by three [1] for the out-of-hospital RSV-attributable deaths. All simulations and analyses were performed in R software (version 4.0.2.) [26].

## Results

### Estimated duration of vaccine-induced immunity

**Fig. 1:**
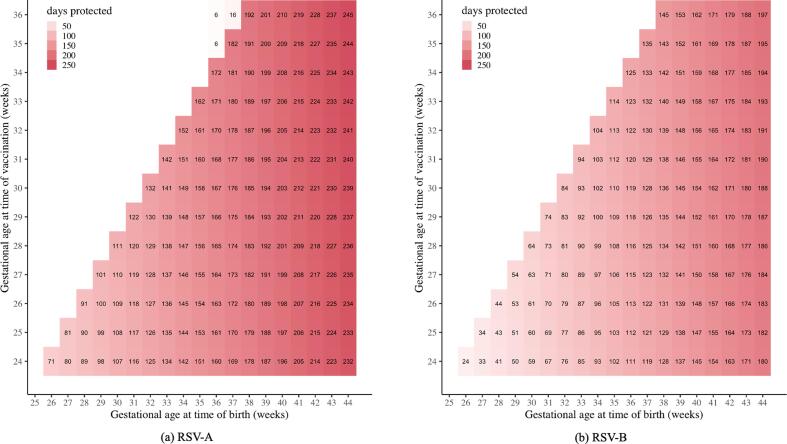
The predicted duration of vaccine-induced immunity (in days) for RSV-A (a) and RSV-B (b) for each combination of gestational age at time of vaccination, and gestational age at time of birth.

We estimated the neonatal RSV-A and RSV-B neutralizing antibody titers at time of birth (Table S3 and Table S4) for each possible combination of gestational age at time of vaccination and gestational age at time of birth (see Methods). Next, we calculated the subsequent duration of vaccine-induced immunity (Fig. 1) for all these combinations.

The model predicts that on average a neonate born at 40 weeks gestational age will be protected for at least 196 days from RSV-A and 145 days from RSV-B related mortality (assuming that vaccination occurred at least 14 days prior to birth) (Fig. 1). The prediction that the vaccine will provide longer protection from RSV-A compared to RSV-B is mostly driven by the higher protection threshold in relation to the prevaccination antibody titers for RSV-B.

For preterms (defined as born before 37 weeks’ gestational age), the duration of vaccine-induced immunity depends strongly on gestational age at time of vaccination and gestational age at time of birth (Fig. 1). The large range in duration of vaccine-induced immunity is mainly driven by a low antibody transfer early in pregnancy that quickly increases with gestational age. Lower antibody transfer at time of birth results in lower antibody titers and therefore a shorter duration of protection.

We applied our model in a situation without vaccination to estimate the duration of natural immunity. The model predicts no protection from RSV-A and RSV-B related mortality for preterm neonates born 35 weeks’ gestational age or younger (Table S5). For full-term neonates, the protection ranges from 16 to 86 days (RSV-A) and from 0 to 29 days (RSV-B), dependent on gestational age at time of birth.

### RSV GOLD database

We applied our model to a subset of the RSV GOLD database [22], including all in-hospital deaths and out-of-hospital deaths from infants in LMICs who died before 6 months of age. The median age at time of death was 2.4 months for the in-hospital deaths, and 1.5 months for the out-of-hospital deaths (Table S2, Fig. S4). The median reported gestational age was 38 weeks (IQR: 35, 39) for the in-hospital deaths, and 38 weeks (IQR: 38, 40) for the out-of-hospital deaths (Table S2, Fig. S4). For the subset with the most reliable observations for gestational age (see Methods), the median gestational age was 38 weeks (IQR: 35, 39).

### Potential impact of maternal vaccination

**Fig. 2:**
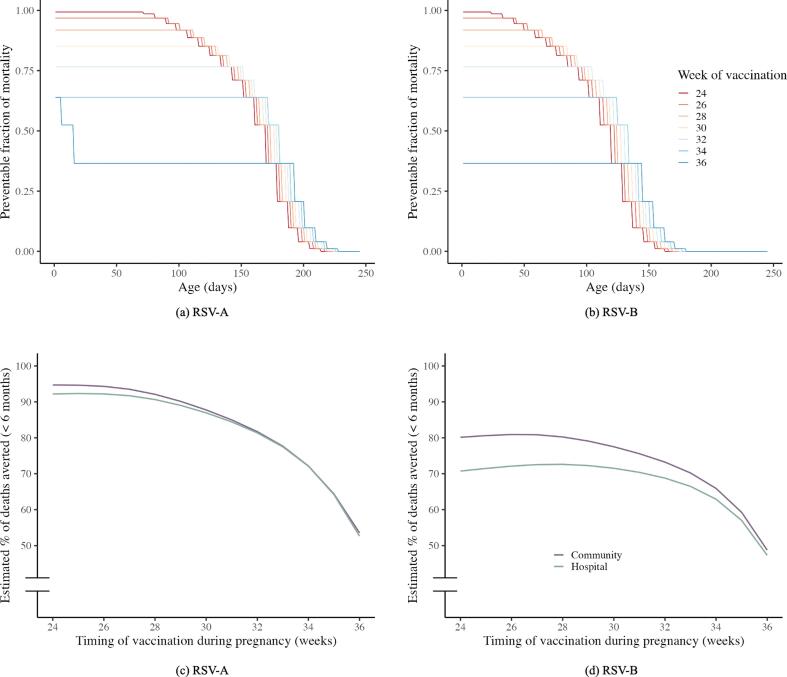
Estimated potential impact of maternal vaccination on RSV-related mortality. Plot (a) and (b) show fraction of mortality cases for RSV-A (a) and RSV-B (b) that could be prevented for the different ages of the infants if all mothers would get vaccinated in a specific week (different weeks are shown in different colors). In other words, it shows the percentage of children who are born with vaccine-induced immunity and how this vaccine-induced immunity wanes over time. Here we see the trade-off between vaccinating earlier; increasing the number of individuals who are eligible to receive the vaccine, while the duration of protection will be slightly shorter. Plot (c) and (d) show the estimates for the total percentage of RSV-A (d) and RSV-B (d) related mortality cases averted below 6 months of age for both out-of-hospital deaths (purple) and in-hospital deaths (green) given timing of vaccination, assuming that all mothers eligible for vaccination received a vaccine.

By considering the distribution of gestational age at the time of birth from the mortality cases in the RSV GOLD database, we made predictions on the proportion of infants who possess vaccine-induced immunity (i.e. their antibody concentration being above the protection threshold) at different ages (Fig. 2a and Fig. 2b). The fraction of prevented mortality is higher when vaccination takes place earlier during pregnancy as it is most likely that the vaccine will have less (or no) effect if the mother delivers soon after the vaccine administration or even before being vaccinated. On the other hand, if vaccination occurs too early during pregnancy, the prevented fraction begins to decline at a slightly younger age; because we assume that maternal antibody concentration starts to decrease 14 days after vaccination in the mother. Consequently, this leads to slightly lower antibody levels at the time of birth for full-term babies. Despite the slightly lower antibody levels at the time of birth for full-term babies, the advantages of vaccinating earlier in pregnancy far outweigh this downside.

Taking both the age at time of death and gestational age at time of birth distributions into account, we can predict vaccine impact in children younger than 6 months in LMICs. For RSV-A, the predicted vaccine-induced duration of protection exceeds 6 months for almost all combinations of gestational age at time of birth and gestational age at time of vaccination (Fig. 1), hence providing almost full protection to both in-hospital and out-of-hospital deaths (Fig. 2). For RSV-B this is not the case; as the predicted vaccine-induced duration of protection is shorter, the potential vaccine impact of the vaccine depends more on the specific age at time of death distribution, causing different impact estimates for in-hospital and out-of-hospital deaths.

### Optimal timing of vaccination

The optimal timing of vaccination depends on the relation between gestational age and antibody transfer over the placenta, the gestational age at time of birth distribution in the population of interest, and the half-life of vaccine induced antibodies in the mother.

For RSV-A, the model predicts that vaccination at 25 weeks’ (in-hospital mortality) and 24 weeks’ (out-of-hospital mortality) gestational age would be optimal (Fig. 2c, Table S6 and Table S7). For RSV-B, the model predicts that vaccination at 27 weeks’ (in-hospital mortality) and 26 weeks’ (out-of-hospital mortality) gestational age would be optimal (Fig. 2d, Table S8 and Table S9).

The predicted optimal timing of vaccination is largely driven by the slow decay of vaccine induced antibodies in the mother for the RSVpreF vaccine (an estimated half-life of 274.5 for RSV-A and 229.5 for RSV-B). When we parameterized the model to the Novavax vaccine characteristics [17], with a half-life of vaccine induced antibodies in the mother of 145.6 days, we found that the optimal timing of vaccination was between 31 and 32 weeks’ gestational age (data not shown).

### Country-specific estimates for vaccination impact

**Fig. 3:**
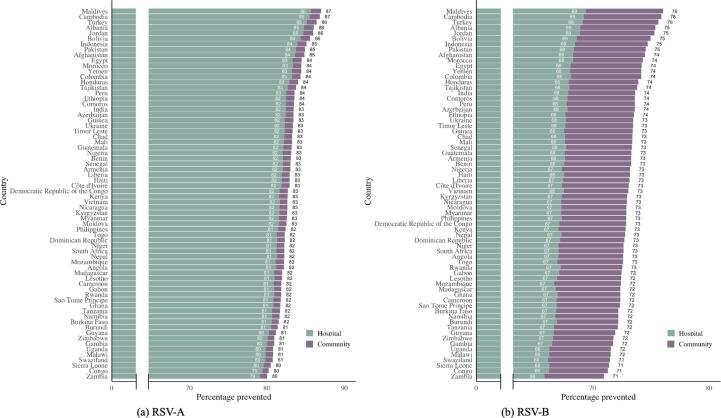
Country-specific estimates for impact of maternal vaccination on RSV-A (a) and RSV-B (b) related mortality, assuming full vaccine coverage.

**Fig. 4:**
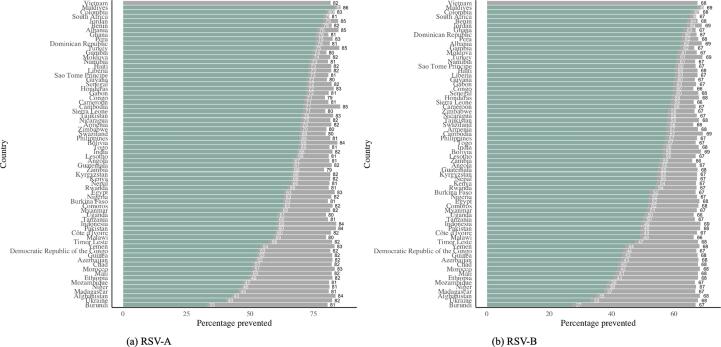
Country-specific estimates for impact of maternal vaccination on RSV-A (a) and RSV-B (b) related in-hospital mortality, assuming full vaccine coverage (grey) and a realistic vaccine coverage (green).

**Fig. 5:**
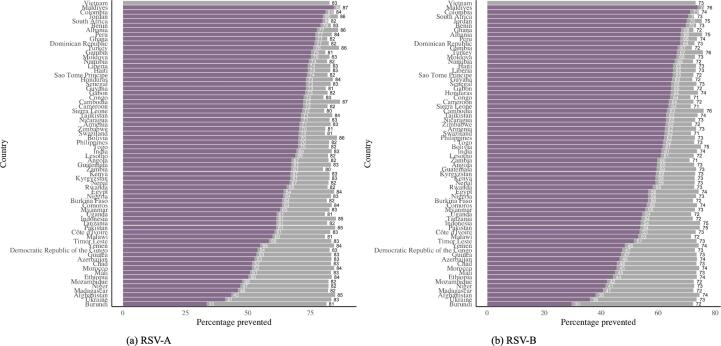
Country-specific estimates for impact of maternal vaccination on RSV-A (a) and RSV-B (b) related out-of-hospital mortality, assuming full vaccine coverage (grey) and a realistic vaccine coverage (purple).

We assumed that antenatal care (ANC) visits could be used as opportunities to administer a maternal RSV vaccine. We used the country-specific distribution of time of ANC visits within the administration window as a proxy for the likelihood distribution for timing of maternal vaccination. The country-specific estimates for vaccine efficacy (Fig. 3) were multiplied by the estimated service availability and acceptance proxy reported by Baral et al. [24]. This yields country-specific estimates for vaccine efficacy, assuming a more realistic vaccine coverage (Fig. 4 for in-hospital mortality and Fig. 5 for out-of-hospital mortality)

Lastly, we estimated for 51 GAVI-eligible countries the number of RSV-related mortality cases averted yearly, using the mortality estimates from Li et al. [25]. We estimated that for those 51 countries, between 17,375 and 15,062 in-hospital mortality cases total (estimate based on vaccine efficacy for RSV-A and RSV-B respectively) could be averted yearly (Table S11). This corresponds to a weighted average 63% of the RSV-A related deaths and 55% of the RSV-B related deaths averted in the selected countries.

## Discussion

We have refined and extended a previously published mathematical model [10] to estimate vaccine efficacy, defined as the percentage of averted RSV-related mortality cases before 6 months. The model was calibrated using the phase 1/2 RSV prefusion F (RSVpreF) vaccine trial [11]. It was applied to simulated data sets based on the observed distributions of gestational age at time of birth and age at time of death in the RSV GOLD database. According to our model, the probability that RSV mortality before 6 months of age is averted by the RSVpreF vaccine depends strongly on gestational age at time of birth. Assuming that early vaccination is deemed entirely safe concerning preterm birth, the most favorable timing for administering the RSVpreF vaccine would be early in pregnancy. From an implementation perspective, it may be crucial to consider the timing of maternal vaccines’ administration in a way that balances optimal antibody response timing while also ensuring maximum vaccine coverage or maximum coverage of other concurrently delivered interventions.

The model predicted that given a probable vaccination coverage, the RSVpreF Vaccine, that recently received approval from the EMA, could decrease the yearly number of RSV-related cases in 51 GAVI-eligible countries in the first half year of life by 15,000–17,000. This corresponds to a weighted average of 55%–63% of all RSV-related mortality cases in the first year. Future research should translate these findings into cost-effectiveness analyses to guide policy makers with prioritizing interventions that have the highest impact and best value for money.

In our previous work [10], we estimated a mortality reduction range of 29%–48% in the global mortality cohort based on the Novavax vaccine, which is notably lower compared to our current estimates. However, it should be noted that the Novavax vaccine, as evaluated in the NCT02624947 phase 3 trial [17], did not meet its primary endpoint of medically significant RSV lower respiratory tract infection (LRTI) and clinical trials were not pursued. To the best of our knowledge, this is the first mathematical modelling study predicting the potential impact of the promising RSVpreF Vaccine in LMICs. Our estimates are in line with data from the phase 2b and phase 3 RSV PreF trial [18], [12], where data on infant neutralizing titers suggest that the maternal vaccine has the potential to protect infants from RSV infection well into their first 6 months of life [18], [12]. The phase 3 RSV PreF trial efficacy analysis showed efficacy of 76.9% for severe medically attended RSV-associated lower respiratory tract illness during the first 180 days in LMICs [27]. Considering that RSV-B was predominant during the trial, our estimates for potential impact in LMICs for a vaccination window between 24 and 36 weeks are in line with the phase 3 trial efficacy estimates. The vaccine efficacy estimates for RSV-A specifically in the phase 3 trial are notably lower compared to the estimates from our model, however these estimates are surrounded by large confidence intervals due to a limited number of RSV-A cases in the phase 3 trial and might therefore be less reliable.

In our model we fitted the antibody transfer function on observed cord blood to maternal ratio IgG levels as described previously [10], as this is the best available data to date. However, besides gestational age, the rate of antibody transfer also depends on the type and subclass of the antibody [28], [29], [30], [31] as well as vaccine formulation and population of interest [17], [18]. Importantly, our predicted antibody transfer ratios, and subsequent fetal antibody levels at time of birth, are lower than observed in the RSVpreF phase 2b trial (where transfer ratios ranged from 1.41 to 2.10) [18], leading to more conservative vaccine impact estimates. In order to improve the prediction of the impact of maternal vaccination, especially for preterm infants, and to improve the prediction of the optimal timing of RSV immunization in pregnancy it is essential to have more granular data evaluating the antibody transfer by week of gestational age [32], [33].

Another important assumption in our model is the choice for the protection threshold. We assumed that palivizumab serum titers, or RSV neutralizing titers corresponding to palivizumab serum titers, equal or above 100 µ*g*/*ml* could avert (or at least postpone) RSV-related mortality. Although this threshold has been the best established correlation with protection to date [34], [35], [11], studies that have investigated other immunological correlates of protections against severe RSV disease have yielded inconsistent findings [36]. As we use the protection threshold to predict the number of days protected, a lower or higher threshold would lead to a under- or overestimation of the number of days protected and therefore different impact estimates. Having a better knowledge on correlates of protection is essential to improve the prediction of the impact of maternal vaccination. Lastly, we assumed that the neonatal antibody half-life of the maternally derived RSV specific antibodies is independent on time, prematurity, weight, or other biological factors. There are some indications that the antibody half-life might be shorter in preterms and in the first 4 months of life [37].

A strength of our study is the use of characteristics of a maternal RSV vaccine currently in development [23] and data simulation based on real data from a global mortality registry [22]. Our model has multiple applications. Firstly, it can be applied to estimate the duration of protection provided by maternal vaccination on case-level, based on gestational age at time of vaccination and gestational age at time of birth. As little research has been carried out on the efficacy of maternal vaccines in prematurely born infants [8], our model can contribute to inform at which gestational age we can assume that infants are protected when born prematurely and contribute to bridge vaccine efficacy to this population. Secondly, our model can be applied to estimate vaccine efficacy on a group or population-level to bridge vaccine efficacy to populations not included in initial efficacy trial(s). Thirdly, our model can help to inform optimal timing of RSV immunization in pregnancy. Lastly, after reparameterization, our model can be applied to other vaccines that can be recommended during pregnancy such as tetanus, diphtheria, pertussis, and influenza, as maternal neutralizing antibodies against these pathogens are also transferred to the fetus during pregnancy [38].

One limitation of our study is the reporting bias, quality, and completeness of the RSV GOLD database, which have been described elsewhere [20], [21], [22]. In our study, gestational age was often missing and was considered unreliable in the LMIC subset in our database. We simulated data based on the most reliable gestational age at time of birth distribution across the full RSV database, including high income countries. Furthermore, for the data simulation we assumed there is no relationship between gestational age at time of birth and age at time of death. Although we did not observe a significant correlation in the RSV GOLD subset, it is not unlikely that a correlation exists, which would lead to bias in our estimates. In addition, for simplicity, and due to a lack of data, we did not incorporate country-specific distributions for age at time of death and gestational age. Our model does not account for comorbidity or other biological factors that may impair antibody transfer, such as low birthweight, maternal HIV infection, and placental malaria [32], despite their high prevalence in LMICs. We did not incorporate breastfeeding into our model, as research on orally administered antibodies has yielded no significant increase in serum titers of intact antibodies [39]. It should also be noted that the estimates for timing of ANC visits in LMICs by gestational age that we used in our model, were based on data published between 2001 and 2018 [24] and might therefore be dated. Furthermore, there is a large uncertainty around the used service availability and acceptance proxy and mortality burden estimates used to make country-specific predictions. For the estimated number of cases averted we assumed a 1:3 in-hospital to out-of-hospital mortality ratio as a crude estimate. However, in a different analysis the estimate of RSV mortality rate has been estimated to be 7- to 10-fold higher out-of-hospital compared with the in-hospital death [40]. Assuming a 1:10 in-hospital to out-of-hospital mortality ratio would change our results to between 47,879 and 41,956 in-hospital mortality cases total (estimate based on vaccine efficacy for RSV-A and RSV-B respectively) averted yearly in those 51 Gavi-eligible countries.

## Conclusion

We predicted that maternal vaccination against RSV could substantially decrease the number of RSV related in-hospital and out-of-hospital deaths before 6 months of age in LMICs. Our model can be used to estimate duration of protection provided by maternal vaccination on case-level and to help inform policy makers on optimal timing of maternal vaccination during pregnancy. It can give an indication of the population-level effectiveness of new interventions prior to introducing a preventive strategy, which is critical for governments and large funding organizations.

## Funding

10.13039/100000865Bill & Melinda Gates Foundation [OPP1148988.8].

## Declaration of Competing Interest

The authors declare the following financial interests/personal relationships which may be considered as potential competing interests: LB has regular interaction with pharmaceutical and other industrial partners. He has not received personal fees or other personal benefits. UMCU has received major funding (>€100,000 per industrial partner) for investigator initiated studies from AbbVie, MedImmune, AstraZeneca, Sanofi, Janssen, Pfizer, MSD, and MeMed Diagnostics. UMCU has received major funding for the RSV GOLD study from the Bill and Melinda Gates Foundation. UMCU has received major funding as part of the public private partnership IMI-funded RESCEU and PROMISE projects with partners GSK, Novavax, Janssen, AstraZeneca, Pfizer, and Sanofi. UMCU has received major funding by Julius Clinical for participating in clinical studies sponsored by MedImmune and Pfizer. UMCU received minor funding (€1,000–25,000 per industrial partner) for consultation and invited lectures by AbbVie, MedImmune, Ablynx, Bavaria Nordic, MabXience, GSK, Novavax, Pfizer, Moderna, Astrazeneca, MSD, Sanofi, Genzyme, Janssen. Dr. Bont is the founding chairman of the ReSViNET Foundation.

Since April 1st 2021, LB has been given a new position as medical scientific division manager of the Children's Division of the Wilhelmina Children's Hospital in Utrecht.

## Data Availability

Data will be made available on request.

## References

[1] Li Y., Wang X., Blau D.M., Caballero M.T., Feikin D.R., Gill C.J. (2022). Global, regional, and national disease burden estimates of acute lower respiratory infections due to respiratory syncytial virus in children younger than 5 years in 2019: a systematic analysis. Lancet.

[2] Mazur N.I., Terstappen J., Baral R., Bardají A., Beutels P., Buchholz U.J. (2022). Respiratory syncytial virus prevention within reach: the vaccine and monoclonal antibody landscape. Lancet Infect Dis.

[3] Mezei A., Cohen J., Renwick M.J., Atwell J., Portnoy A. (2021). Mathematical modelling of respiratory syncytial virus (RSV) in low-and middle-income countries: a systematic review. Epidemics.

[4] Li Y., Hodgson D., Wang X., Atkins K.E., Feikin D.R., Nair H. (2021). Respiratory syncytial virus seasonality and prevention strategy planning for passive immunisation of infants in low-income and middle-income countries: a modelling study. Lancet Infect Dis.

[5] Giersing BK, Karron RA, Vekemans J, Kaslow DC, Moorthy VS. Meeting report: Who consultation on respiratory syncytial virus (RSV) vaccine development, Geneva, 25–26 April 2016. Vaccine 2019;37(50):7355–62. 10.1016/j.vaccine.2017.02.068 28302410

[6] Kampmann B., Madhi S.A., Munjal I., Simões E.A., Pahud B.A., Llapur C. (2023). Bivalent prefusion F vaccine in pregnancy to prevent RSV illness in infants. N Engl J Med.

[7] Ema. First RSV vaccine to protect infants up 6 months of age and older adults; 2023. URL: https://www.ema. europa.eu/en/news/first-RSV-vaccine-protect-infants-6-months-age-older-adults.

[8] Phijffer EW, Bont LJ. Are we ready for maternal respiratory syncytial virus vaccination? 2022. 10.1093/infdis/jiab613 34932123

[9] Baral R., Li X., Willem L., Antillon M., Vilajeliu A., Jit M. (2020). The impact of maternal RSV vaccine to protect infants in gavi-supported countries: estimates from two models. Vaccine.

[10] Scheltema N.M., Kavelaars X.M., Thorburn K., Hennus M.P., van Woensel J.B., van der Ent C.K. (2018). Potential impact of maternal vaccination on life-threatening respiratory syncytial virus infection during infancy. Vaccine.

[11] Walsh E.E., Falsey A.R., Scott D.A., Gurtman A., Zareba A.M., Jansen K.U. (2022). A randomized phase 1/2 study of a respiratory syncytial virus prefusion F vaccine. J Infect Dis.

[12] U.S. FDA accepts biologics license application for Pfizer’s respiratory syncytial virus maternal vaccine candidate for priority review 2023; URL: https://www.pfizer.com/news/press-release/press-release-detail/us-fda-accepts-biologics-license-application-pfizers.

[13] Halperin B., Morris A., Mackinnon-Cameron D., Mutch J., Langley J., McNeil S. (2011). Kinetics of the antibody response to tetanus-diphtheria-acellular pertussis vaccine in women of childbearing age and postpartum women. Clin Infect Dis.

[14] Ruckwardt T.J., Morabito K.M., Phung E., Crank M.C., Costner P.J., Holman L.A. (2021). Safety, tolerability, and immunogenicity of the respiratory syncytial virus prefusion f subunit vaccine ds-cav1: a phase 1, randomised, open-label, dose-escalation clinical trial. Lancet Respir Med.

[15] Glenn G.M., Fries L.F., Thomas D.N., Smith G., Kpamegan E., Lu H. (2016). A randomized, blinded, controlled, dose-ranging study of a respiratory syncytial virus recombinant fusion (f) nanoparticle vaccine in healthy women of childbearing age. J Infect Dis.

[16] Falsey A.R., Walsh E.E., Scott D.A., Gurtman A., Zareba A., Jansen K.U. (2022). Phase 1/2 randomized study of the immunogenicity, safety, and tolerability of a respiratory syncytial virus prefusion F vaccine in adults with concomitant inactivated influenza vaccine. J Infect Dis.

[17] Madhi S.A., Polack F.P., Piedra P.A., Munoz F.M., Trenholme A.A., Simões E.A. (2020). Respiratory syncytial virus vaccination during pregnancy and effects in infants. N Engl J Med.

[18] Simões E.A., Center K.J., Tita A.T., Swanson K.A., Radley D., Houghton J. (2022). Prefusion F protein–based respiratory syncytial virus immunization in pregnancy. N Engl J Med.

[19] Muňoz F.M., Swamy G.K., Hickman S.P., Agrawal S., Piedra P.A., Glenn G.M. (2019). Safety and immunogenicity of a respiratory syncytial virus fusion (F) protein nanoparticle vaccine in healthy third-trimester pregnant women and their infants. J Infect Dis.

[20] Scheltema N.M., Gentile A., Lucion F., Nokes D.J., Munywoki P.K., Madhi S.A. (2017). Global respiratory syncytial virus-associated mortality in young children (RSV gold): a retrospective case series. Lancet Global Health.

[21] Löwensteyn Y.N., Phijffer E.W., Simons J.V., Scheltema N.M., Mazur N.I., Nair H. (2020). Respiratory syncytial virus-related death in children with down syndrome: the RSV gold study. Pediatr Infect Dis J.

[22] Mazur N.I., Löwensteyn Y.N., Willemsen J.E., Gill C.J., Forman L., Mwananyanda L.M. (2021). Global respiratory syncytial virus–related infant community deaths. Clin Infect Dis.

[23] GOV C. A trial to evaluate the efficacy and safety of RSVpref in infants born to women vaccinated during pregnancy; 2021.

[24] Baral R., Fleming J., Khan S., Higgins D., Hendrix N., Pecenka C. (2020). Inferring antenatal care visit timing in low-and middle-income countries: methods to inform potential maternal vaccine coverage. PLoS ONE.

[25] Li X., Willem L., Antillon M., Bilcke J., Jit M., Beutels P. (2020). Health and economic burden of respiratory syncytial virus (RSV) disease and the cost-effectiveness of potential interventions against RSV among children under 5 years in 72 gavi-eligible countries. BMC Med.

[26] R Core Team. R: A Language and Environment for Statistical Computing. Vienna, Austria: R Foundation for Statistical Computing; 2021. URL: https://www.R-project.org/.

[27] Kampman B. Safety and efficacy of bivalent RSV prefusion F vaccine in vaccinated mothers and their infants; 2023. URL: https://www.resvinet.org/session-recordings.html; resvinet Conference 2023.

[28] Ciobanu AM, Dumitru AE, Gica N, Botezatu R, Peltecu G, Panaitescu AM. Benefits and risks of igg transplacental transfer. Diagnostics 2020;10(8):583. 10.3390/diagnostics10080583 PMC7459488 32806663

[29] Jennewein MF, Goldfarb I, Dolatshahi S, Cosgrove C, Noelette FJ, Krykbaeva M, et al. FC glycan-mediated regulation of placental antibody transfer. Cell 2019;178(1):202–15. 10.1016/j.cell.2019.05.044 PMC6741440 31204102

[30] Palmeira P, Quinello C, Silveira-Lessa AL, Zago CA, Carneiro-Sampaio M. Igg placental transfer in healthy and pathological pregnancies. Clin Dev Immunol 2012;2012. 10.1155/2012/985646 PMC3251916 22235228

[31] Clements T, Rice TF, Vamvakas G, Barnett S, Barnes M, Donaldson B, et al. Update on transplacental transfer of igg subclasses: impact of maternal and fetal factors. Front Immunol 2020:1920. 10.3389/fimmu.2020.01920 PMC7516031 33013843

[32] Atwell J.E., Lutz C.S., Sparrow E.G., Feikin D.R. (2022). Biological factors that may impair transplacental transfer of RSV antibodies: implications for maternal immunization policy and research priorities for low-and middle-income countries. Vaccine.

[33] Fouda G.G., Martinez D.R., Swamy G.K., Permar S.R. (2018). The impact of igg transplacental transfer on early life immunity. Immunohorizons.

[34] Hervé P.L., Dhelft V., Zuniga A., Ghasparian A., Rassek O., Yim K.C. (2021). Epicutaneous immunization using synthetic virus-like particles efficiently boosts protective immunity to respiratory syncytial virus. Vaccine.

[35] Forbes M.L., Kumar V.R., Yogev R., Wu X., Robbie G.J., Ambrose C.S. (2014). Serum palivizumab level is associated with decreased severity of respiratory syncytial virus disease in high-risk infants. Hum Vaccin Immunother.

[36] Abu-Raya B., Reicherz F., Lavoie P.M. (2022). Correlates of protection against respiratory syncytial virus infection in infancy. Clin Rev Allergy Immunol.

[37] Nyiro J.U., Sande C., Mutunga M., Kiyuka P.K., Munywoki P.K., Scott J.A.G. (2015). Quantifying maternally derived respiratory syncytial virus specific neutralising antibodies in a birth cohort from coastal Kenya. Vaccine.

[38] Albrecht M., Arck P.C. (2020). Vertically transferred immunity in neonates: mothers, mechanisms and mediators. Front Immunol.

[39] Reilly R.M., Domingo R., Sandhu J. (1997). Oral delivery of antibodies. Clin Pharmacokinet.

[40] Simões EA, Dani V, Potdar V, Crow R, Satav S, Chadha MS, et al. Mortality from respiratory syncytial virus in children under 2 years of age: a prospective community cohort study in rural Maharashtra, India. Clin Infect Dis 2021;73(Suppl._3):S193–202. 10.1093/cid/ciab481 PMC8411248 34472578

