# Supplementary material for: Maternal vaccination against RSV can substantially reduce childhood mortality in low-income and middle-income countries: A mathematical modeling study
Source: Vaccine X. 2023 Sep 1;15:100379. doi: 10.1016/j.jvacx.2023.100379 (PMC10498305; doi:10.1016/j.jvacx.2023.100379)
Supplement: Supplementary data 1 [file mmc1.docx]

# S1. Simulated age and gestational age distributions

The age (in days) at time of death distribution in the population is needed to predict the potential impact of maternal vaccination. Unfortunately, this information is reported in months instead of days in a large part of the dataset. Age at time of death was reported in months for 65.1% (308/473) of the cases for the in-hospital deaths, and 9.0% (14/156) of the cases for the out-of-hospital deaths, Figure [S5a](#_bookmark75)). We expected that simply converting months to days would result in rounding errors and misclassification bias. We therefore decided to simulate data distributions to create more representative data, see appendix [S1](#_bookmark50) for more details.

For gestational age, information on gestational age was missing in a large proportion of cases (74.2% for the in-hospital deaths and 85.3% for the out-of-hospital deaths). Furthermore, we noticed an over-representation of infants born at full-term, specifically infants born at 38 weeks gestational age. We expected that the reported gestational age distribution was not representative of the true population due to response and reporting bias. To fill in the data gaps and create a more representative distribution, we used a subset of the most reliable observations (when gestational age was estimated based on either last menstrual period or ultrasonography) of the reported gestational ages in the entire RSV GOLD database (Figure [S6a](#_bookmark77)), see appendix [S1](#_bookmark50) for more details.

Firstly, cases where age at time of death was reported in months were resampled (Figure [S5b](#_bookmark75)). To account for miss-classification bias, we also included cases that were reported to be six months of age (reported in months, not days), yielding an additional 30 in-hospital death and 1 out-of-hospital death. We assumed that age was rounded to the nearest integer when reported to the RSV GOLD database, using a uniform distribution:

$$x_{age} \sim UNIF(x_{age}- \frac{1}{2}, x_{age}+ \frac{1}{2} )$$

(A.1)

Secondly, based on the age reported in days and the re-sampled values taken together, we fitted a smoothed distribution using kernel density estimation using the function ”density” in R, with the default bandwith and 2^14^ number of equally spaced points at which the density is estimated. The distribution was then truncated to exclude *x_age_* ≥ 182 days (Figure [S5c](#_bookmark75)). This process was repeated a 1000 times to account for sampling uncertainty, starting from re-sampling the age reported in months. This yields a 1000 simulated age at time of death distributions for both out-of-hospital deaths and in-hospital deaths.

As gestational age is reported in full weeks in the database, we used the function ”dkde1d” in package ”kde1d” [[1](#_bookmark48)] to estimate the smoothed distribution using kernel density estimation, as it can handle discrete data [[1](#_bookmark48)] (Figure [S6b](#_bookmark77)). There is minimal variation in the predicted percentage prevented for each partly re-sampled age at time of death distribution. The median predicted percentage prevented per time of vaccination and range across simulations are reported in appendix [S3.4](#_bookmark60).

# S2. Estimating the half-life of vaccine induced antibodies in the mother

Assuming that the maternal RSV-A and RSV-B neutralizing antibody levels follow an exponential decline after vaccination, we can estimate the half-life of the neutralizing antibodies based on the reported geometric mean fold ratio (GMFR) measurements at two different time points. We took the measurement at the first month after vaccination and 12th month after vaccination as reported in Figure 4 of [2]. We estimated the half-life using the following equation:

$$t_{M_{\frac{1}{2}}}=(t_{m1}- t_{m2})/(-log(M2/M1)/log(2))$$

(B.1)

.

Where *t_m_*_1_ and *t_m_*_1_ are the first and second time point in days (30.4 and 12 ∗ 30.4). *M*1 is the measurement at the first time point (12.1 for RSV-A and 14.0 for RSV-B) and *M*2 is the measurement at the second time point (5.2 for RSV-A and 5.1 for RSV-B). This yields a half-life of $t_{M_{1/2}}$= 274.5 for RSV-A and $t_{M_{1/2}}$ = 229.5 for RSV-B.

For fold increase at 14 days post vaccination, we used the half-life formula to back-extrapolate antibody titers between day 14 ( *f_t_*_14_) and day 31 ( *f_t_*_31_) post-vaccination based on the reported fold increase at day 31 and the calculated the half-life:

$$f_{t31}= f_{t14}*{(1/2)}^{(31-14)/t_{m_{\frac{1}{2}}}}$$

This yielded a fold increase of 12.6 for RSV-A and 14.7 for RSV-B 14 days post vaccination.

# S3. Supplementary Tables

*S3.1. RSV GOLD database*

Table S2: Clinical characteristics in infants below 6 months of age from LMICs in the RSV GOLD database [4].

|  | out-of-hospital deaths | in-hospital deaths |
| --- | --- | --- |
|  | (n = 156) | (n = 473) |
| Age at time of death, median (IQR) | 1.5 (0.8, 3.3) | 2.4 (1.5, 4.0) |
| Gestational age, median (IQR) | 38 (38, 40) | 38 (35, 39) |
| Prematurity, % (n/N) | 25 (11/44) | 32 (81/253) |
| Comorbidity, % (n/N) | 29 (10/35) | 49 (179/368) |
| Sex, % (n/N) | 53 (78/142) | 55 (78/142) |
| Country of origin |  |  |
| Low income | 5 | 34 |
| Lower middle income | 138 | 187 |
| Upper middle income | 13 | 252 |

*S3.2. Predicted antibody levels at time of birth*

Table S3: Predicted antibody levels at time of birth for RSV-A

|  | 24 | 25 | 26 | 27 | 28 | 29 | 30 | 31 | 32 | 33 | 34 | 35 | 36 |
| --- | --- | --- | --- | --- | --- | --- | --- | --- | --- | --- | --- | --- | --- |
| 24 | 156 |  |  |  |  |  |  |  |  |  |  |  |  |
| 25 | 182 | 182 |  |  |  |  |  |  |  |  |  |  |  |
| 26 | 2644 | 212 | 212 |  |  |  |  |  |  |  |  |  |  |
| 27 | 3045 | 3083 | 247 | 247 |  |  |  |  |  |  |  |  |  |
| 28 | 3508 | 3551 | 3595 | 288 | 288 |  |  |  |  |  |  |  |  |
| 29 | 4041 | 4091 | 4141 | 4192 | 336 | 336 |  |  |  |  |  |  |  |
| 30 | 4655 | 4713 | 4771 | 4829 | 4889 | 392 | 392 |  |  |  |  |  |  |
| 31 | 5363 | 5429 | 5496 | 5563 | 5632 | 5701 | 457 | 457 |  |  |  |  |  |
| 32 | 6178 | 6254 | 6331 | 6409 | 6487 | 6567 | 6648 | 533 | 533 |  |  |  |  |
| 33 | 7116 | 7204 | 7293 | 7382 | 7473 | 7565 | 7658 | 7753 | 622 | 622 |  |  |  |
| 34 | 8198 | 8299 | 8401 | 8504 | 8609 | 8715 | 8822 | 8931 | 9041 | 725 | 725 |  |  |
| 35 | 9443 | 9560 | 9677 | 9796 | 9917 | 10039 | 10163 | 10288 | 10414 | 10542 | 846 | 846 |  |
| 36 | 10878 | 11012 | 11148 | 11285 | 11424 | 11564 | 11707 | 11851 | 11997 | 12144 | 12294 | 986 | 986 |
| 37 | 12531 | 12685 | 12842 | 13000 | 13160 | 13322 | 13486 | 13652 | 13820 | 13990 | 14162 | 14336 | 1150 |
| 38 | 14435 | 14613 | 14793 | 14975 | 15159 | 15346 | 15535 | 15726 | 15920 | 16115 | 16314 | 16515 | 16718 |
| 39 | 16629 | 16833 | 17041 | 17250 | 17463 | 17678 | 17895 | 18116 | 18339 | 18564 | 18793 | 19024 | 19258 |
| 40 | 19155 | 19391 | 19630 | 19872 | 20116 | 20364 | 20614 | 20868 | 21125 | 21385 | 21648 | 21915 | 22185 |
| 41 | 22066 | 22338 | 22613 | 22891 | 23173 | 23458 | 23747 | 24039 | 24335 | 24635 | 24938 | 25245 | 25555 |
| 42 | 25419 | 25732 | 26049 | 26369 | 26694 | 27023 | 27355 | 27692 | 28033 | 28378 | 28727 | 29081 | 29439 |
| 43 | 29282 | 29642 | 30007 | 30376 | 30750 | 31129 | 31512 | 31900 | 32292 | 32690 | 33092 | 33500 | 33912 |
| 44 | 33731 | 34146 | 34566 | 34992 | 35423 | 35859 | 36300 | 36747 | 37199 | 37657 | 38121 | 38590 | 39065 |

s5

Table S4: Predicted antibody levels at time of birth for RSV-B

s6

|  | 24 | 25 | 26 | 27 | 28 | 29 | 30 | 31 | 32 | 33 | 34 | 35 | 36 |
| --- | --- | --- | --- | --- | --- | --- | --- | --- | --- | --- | --- | --- | --- |
| 24 | 149 |  |  |  |  |  |  |  |  |  |  |  |  |
| 25 | 173 | 173 |  |  |  |  |  |  |  |  |  |  |  |
| 26 | 2931 | 202 | 202 |  |  |  |  |  |  |  |  |  |  |
| 27 | 3368 | 3418 | 236 | 236 |  |  |  |  |  |  |  |  |  |
| 28 | 3871 | 3928 | 3985 | 275 | 275 |  |  |  |  |  |  |  |  |
| 29 | 4448 | 4514 | 4580 | 4648 | 320 | 320 |  |  |  |  |  |  |  |
| 30 | 5112 | 5187 | 5263 | 5341 | 5420 | 374 | 374 |  |  |  |  |  |  |
| 31 | 5874 | 5961 | 6049 | 6138 | 6228 | 6320 | 436 | 436 |  |  |  |  |  |
| 32 | 6751 | 6850 | 6951 | 7054 | 7158 | 7263 | 7370 | 508 | 508 |  |  |  |  |
| 33 | 7758 | 7872 | 7988 | 8106 | 8225 | 8347 | 8470 | 8594 | 592 | 592 |  |  |  |
| 34 | 8915 | 9047 | 9180 | 9315 | 9453 | 9592 | 9733 | 9877 | 10022 | 691 | 691 |  |  |
| 35 | 10246 | 10397 | 10550 | 10705 | 10863 | 11023 | 11185 | 11350 | 11517 | 11687 | 806 | 806 |  |
| 36 | 11774 | 11948 | 12124 | 12302 | 12484 | 12668 | 12854 | 13044 | 13236 | 13431 | 13629 | 940 | 940 |
| 37 | 13531 | 13730 | 13933 | 14138 | 14346 | 14558 | 14772 | 14990 | 15211 | 15435 | 15662 | 15893 | 1096 |
| 38 | 15550 | 15779 | 16011 | 16247 | 16487 | 16729 | 16976 | 17226 | 17480 | 17738 | 17999 | 18264 | 18533 |
| 39 | 17870 | 18133 | 18400 | 18671 | 18946 | 19225 | 19509 | 19796 | 20088 | 20384 | 20684 | 20989 | 21298 |
| 40 | 20536 | 20838 | 21145 | 21457 | 21773 | 22094 | 22419 | 22750 | 23085 | 23425 | 23770 | 24121 | 24476 |
| 41 | 23599 | 23947 | 24300 | 24658 | 25021 | 25390 | 25764 | 26144 | 26529 | 26920 | 27317 | 27719 | 28128 |
| 42 | 27120 | 27520 | 27925 | 28337 | 28755 | 29178 | 29608 | 30044 | 30487 | 30936 | 31392 | 31855 | 32324 |
| 43 | 31167 | 31626 | 32092 | 32565 | 33045 | 33532 | 34026 | 34527 | 35036 | 35552 | 36076 | 36607 | 37147 |
| 44 | 35817 | 36344 | 36880 | 37423 | 37975 | 38534 | 39102 | 39678 | 40263 | 40856 | 41458 | 42069 | 42689 |

*S3.3. Predicted duration of vaccine-induced immunity without vaccination*

Table S5: The predicted duration of vaccine-induced immunity (in days) for RSV-A and RSV-B without vaccination, dependent on gestational age at time of birth (GA).

| GA | RSV-A | RSV-B |
| --- | --- | --- |
| 24 | 0 | 0 |
| 25 | 0 | 0 |
| 26 | 0 | 0 |
| 27 | 0 | 0 |
| 28 | 0 | 0 |
| 29 | 0 | 0 |
| 30 | 0 | 0 |
| 31 | 0 | 0 |
| 32 | 0 | 0 |
| 33 | 0 | 0 |
| 34 | 0 | 0 |
| 35 | 0 | 0 |
| 36 | 6 | 0 |
| 37 | 16 | 0 |
| 38 | 26 | 0 |
| 39 | 36 | 0 |
| 40 | 46 | 0 |
| 41 | 56 | 0 |
| 42 | 66 | 9 |
| 43 | 76 | 19 |
| 44 | 86 | 29 |

*S3.4. Predicted percentage prevented range across simulations*

Table S6: Range in predicted vaccine impact for RSV-A Hospital deaths

| Tvac (week) | Min (%) | Median (%) | Max (%) |
| --- | --- | --- | --- |
| 24 | 91.61 | 92.20 | 92.90 |
| 25 | 91.75 | 92.32 | 92.98 |
| 26 | 91.69 | 92.22 | 92.85 |
| 27 | 91.22 | 91.71 | 92.30 |
| 28 | 90.20 | 90.65 | 91.18 |
| 29 | 88.64 | 89.03 | 89.51 |
| 30 | 86.63 | 86.95 | 87.35 |
| 31 | 84.17 | 84.42 | 84.75 |
| 32 | 81.17 | 81.37 | 81.61 |
| 33 | 77.35 | 77.48 | 77.63 |
| 34 | 72.04 | 72.09 | 72.16 |
| 35 | 64.17 | 64.18 | 64.20 |
| 36 | 52.57 | 52.64 | 52.72 |

Tvac = Gestational age at time of vaccination

Table S7: Range in predicted vaccine impact for RSV-A Community deaths

| Tvac (week) | Min (%) | Median (%) | Max (%) |
| --- | --- | --- | --- |
| 24 | 94.37 | 94.71 | 95.04 |
| 25 | 94.32 | 94.65 | 94.96 |
| 26 | 94.02 | 94.32 | 94.61 |
| 27 | 93.23 | 93.51 | 93.79 |
| 28 | 91.84 | 92.10 | 92.35 |
| 29 | 89.91 | 90.14 | 90.37 |
| 30 | 87.55 | 87.76 | 87.97 |
| 31 | 84.81 | 84.98 | 85.16 |
| 32 | 81.58 | 81.72 | 81.85 |
| 33 | 77.57 | 77.66 | 77.74 |
| 34 | 72.12 | 72.16 | 72.19 |
| 35 | 64.33 | 64.34 | 64.35 |
| 36 | 53.51 | 53.55 | 53.59 |

Tvac = Gestational age at time of vaccination

Table S8: Range in predicted vaccine impact for RSV-B Hospital deaths

| Tvac (week) | Min (%) | Median (%) | Max (%) |
| --- | --- | --- | --- |
| 24 | 70.59 | 70.59 | 70.59 |
| 25 | 71.33 | 71.33 | 71.33 |
| 26 | 71.96 | 71.96 | 71.96 |
| 27 | 72.30 | 72.30 | 72.30 |
| 28 | 72.28 | 72.28 | 72.28 |
| 29 | 71.92 | 71.92 | 71.92 |
| 30 | 71.09 | 71.09 | 71.09 |
| 31 | 69.80 | 69.80 | 69.80 |
| 32 | 68.12 | 68.12 | 68.12 |
| 33 | 65.62 | 65.62 | 65.62 |
| 34 | 62.23 | 62.23 | 62.23 |
| 35 | 57.13 | 57.13 | 57.13 |
| 36 | 48.04 | 48.04 | 48.04 |

Tvac = Gestational age at time of vaccination

Table S9: Range in predicted vaccine impact for RSV-B Community deaths

| Tvac (week) | Min (%) | Median (%) | Max (%) |
| --- | --- | --- | --- |
| 24 | 79.45 | 80.14 | 80.84 |
| 25 | 79.92 | 80.61 | 81.32 |
| 26 | 80.22 | 80.91 | 81.63 |
| 27 | 80.16 | 80.85 | 81.56 |
| 28 | 79.57 | 80.24 | 80.93 |
| 29 | 78.45 | 79.09 | 79.76 |
| 30 | 76.89 | 77.51 | 78.16 |
| 31 | 74.98 | 75.58 | 76.21 |
| 32 | 72.66 | 73.24 | 73.85 |
| 33 | 69.67 | 70.22 | 70.80 |
| 34 | 65.40 | 65.91 | 66.47 |
| 35 | 58.79 | 59.26 | 59.76 |
| 36 | 48.37 | 48.74 | 49.14 |

Tvac = Gestational age at time of vaccination

*S3.5. Country-specific estimates for number in-hospital RSV-related mortality cases averted yearly*

| Country | Number of RSV-related deaths, age 0-6 months [[3](#_bookmark46)] | RSV A estimates for number of cases averted | RSV B estimates for number of cases averted |
| --- | --- | --- | --- |
| India | 2377 | 1628 | 1340 |
| Nigeria | 630 | 398 | 327 |
| Pakistan | 542 | 327 | 267 |
| Indonesia | 419 | 254 | 207 |
| Ethiopia | 313 | 155 | 127 |
| The Congo | 292 | 155 | 127 |
| Tanzania | 205 | 124 | 103 |
| Uganda | 170 | 103 | 86 |
| Kenya | 138 | 91 | 75 |
| Mozambique | 107 | 50 | 41 |
| Angola | 106 | 71 | 58 |
| Afghanistan | 100 | 43 | 35 |
| Niger | 95 | 44 | 36 |
| Cameroon | 82 | 58 | 48 |
| Yemen | 82 | 44 | 36 |
| Madagascar | 78 | 35 | 29 |
| Ghana | 76 | 58 | 48 |
| Côte d’Ivoire | 74 | 44 | 36 |
| Burkina Faso | 71 | 45 | 37 |
| Mali | 71 | 36 | 29 |
| Malawi | 65 | 38 | 32 |
| Myanmar | 62 | 38 | 32 |
| Zambia | 62 | 41 | 34 |
| Senegal | 61 | 44 | 36 |
| Chad | 56 | 29 | 23 |
| Nepal | 53 | 35 | 29 |
| Zimbabwe | 45 | 31 | 26 |
| Guinea | 44 | 23 | 19 |
| Ukraine | 43 | 17 | 14 |
| Burundi | 40 | 13 | 11 |
| Benin | 37 | 29 | 24 |
| Cambodia | 32 | 23 | 18 |
| Rwanda | 31 | 20 | 17 |
| Togo | 25 | 17 | 14 |
| Haiti | 22 | 16 | 13 |
| Sierra Leone | 22 | 16 | 13 |
| Tajikistan | 22 | 15 | 13 |
| Bolivia | 21 | 15 | 12 |
| Honduras | 18 | 13 | 11 |
| Congo | 17 | 12 | 10 |
| Azerbaijan | 15 | 8 | 6 |
| Liberia | 14 | 10 | 8 |
| Kyrgyzstan | 13 | 9 | 7 |
| Nicaragua | 10 | 7 | 6 |
| Gambia | 8 | 6 | 5 |
| Lesotho | 5 | 3 | 3 |
| Armenia | 4 | 3 | 2 |
| Moldova | 4 | 3 | 2 |
| Comoros | 3 | 2 | 2 |
| Timor Leste | 3 | 2 | 1 |
| Guyana | 1 | 1 | 1 |
| Total | 6886 | 4302 (62%) | 3536 (51%) |

*S3.6. Country-specific estimates for number out-of-hospital RSV-related mortality cases averted yearly*

| Country | Number of RSV-related deaths, age 0-6 months [[3](#_bookmark46)] | RSV A estimates for number of cases averted | RSV B estimates for number of cases averted |
| --- | --- | --- | --- |
| India | 7131 | 4944 | 4362 |
| Nigeria | 1890 | 1211 | 1066 |
| Pakistan | 1626 | 995 | 874 |
| Indonesia | 1257 | 771 | 677 |
| Ethiopia | 939 | 471 | 415 |
| Democratic Republic |  |  |  |
|  | 876 | 471 | 415 |
| of the Congo |  |  |  |
| Tanzania | 615 | 377 | 333 |
| Uganda | 510 | 313 | 277 |
| Kenya | 414 | 277 | 244 |
| Mozambique | 321 | 153 | 135 |
| Angola | 318 | 214 | 189 |
| Afghanistan | 300 | 130 | 114 |
| Niger | 285 | 134 | 118 |
| Cameroon | 246 | 177 | 157 |
| Yemen | 246 | 135 | 119 |
| Madagascar | 234 | 107 | 95 |
| Ghana | 228 | 175 | 155 |
| Côte d’Ivoire | 222 | 135 | 119 |
| Burkina Faso | 213 | 136 | 120 |
| Mali | 213 | 108 | 95 |
| Malawi | 195 | 117 | 103 |
| Myanmar | 186 | 117 | 103 |
| Zambia | 186 | 125 | 111 |
| Senegal | 183 | 134 | 118 |
| Chad | 168 | 87 | 77 |
| Nepal | 159 | 106 | 94 |
| Zimbabwe | 135 | 95 | 84 |
| Guinea | 132 | 71 | 62 |
| Ukraine | 129 | 53 | 46 |
| Burundi | 120 | 40 | 36 |
| Benin | 111 | 88 | 77 |
| Cambodia | 96 | 69 | 61 |
| Rwanda | 93 | 61 | 54 |
| Togo | 75 | 52 | 46 |
| Haiti | 66 | 49 | 43 |
| Sierra Leone | 66 | 47 | 42 |
| Tajikistan | 66 | 47 | 41 |
| Bolivia | 63 | 44 | 39 |
| Honduras | 54 | 40 | 35 |
| Congo | 51 | 37 | 33 |
| Azerbaijan | 45 | 24 | 21 |
| Liberia | 42 | 31 | 27 |
| Kyrgyzstan | 39 | 26 | 23 |
| Nicaragua | 30 | 21 | 19 |
| Gambia | 24 | 18 | 16 |
| Lesotho | 15 | 10 | 9 |
| Armenia | 12 | 8 | 7 |
| Moldova | 12 | 9 | 8 |
| Comoros | 9 | 6 | 5 |
| Timor Leste | 9 | 5 | 5 |
| Guyana | 3 | 2 | 2 |
| Total | 2065 | 13073 (63%) | 11526 (56%) |

* Estimated out-of-hospital mortality based on in-hospital mortality estimates [3] multiplied by a conversion factor of 3 [[1](#_bookmark8)]

# S4. Supplementary Figures

*S4.1. Predicted percentage of mortality cases averted*

We compared the predicted duration of vaccine-induced immunity after birth with the simulated age at time of death distributions (see Methods) for every possible combination of gestational age at time of vaccination and gestational age at time of birth. This yields the estimated probability that RSV-related death could have been averted (or at least postponed) in infants below 6 months of age for each combination of gestational age at time of vaccination and gestational age at time of birth (Figure [S1](#_bookmark70) and Figure [S2](#_bookmark71)).

The model predicts that on average for a neonate born at 40 weeks gestational age, the probability that RSV-A-related mortality before 6 months would be averted by maternal vaccination is 100% for both in- and out-of-hospital mortality cases (Figure [S1](#_bookmark70) and Figure [S2](#_bookmark71)). For RSV-B-related mortality this probability is between 87% and 94% for in-hospital mortality and between 91% and 96% for out-of-hospital mortality, depending on gestational age at time of vaccination (assuming that the mother had been vaccinated before 36 weeks gestational age).

For preterms, the estimates from 0% to 98% for both in-hospital cases and out-of-hospital cases (assuming that vaccination occurred at least 14 days prior to birth) (Figure [S1](#_bookmark70) and Figure [S2](#_bookmark71)). For RSV-B the estimates range from 0% to 76% for in-hospital cases and from 0% to 84% for out-of-hospital cases.


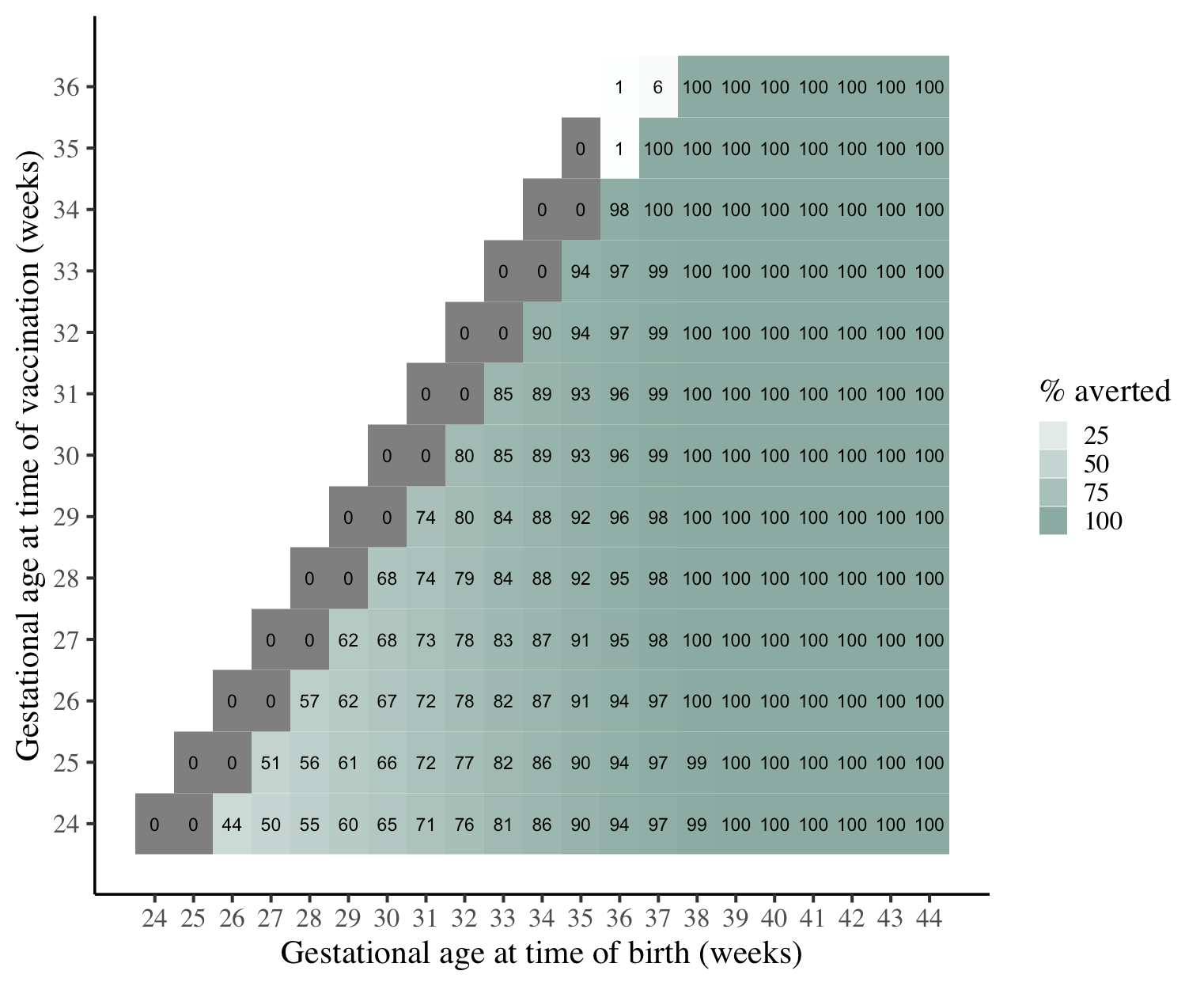


- 1.
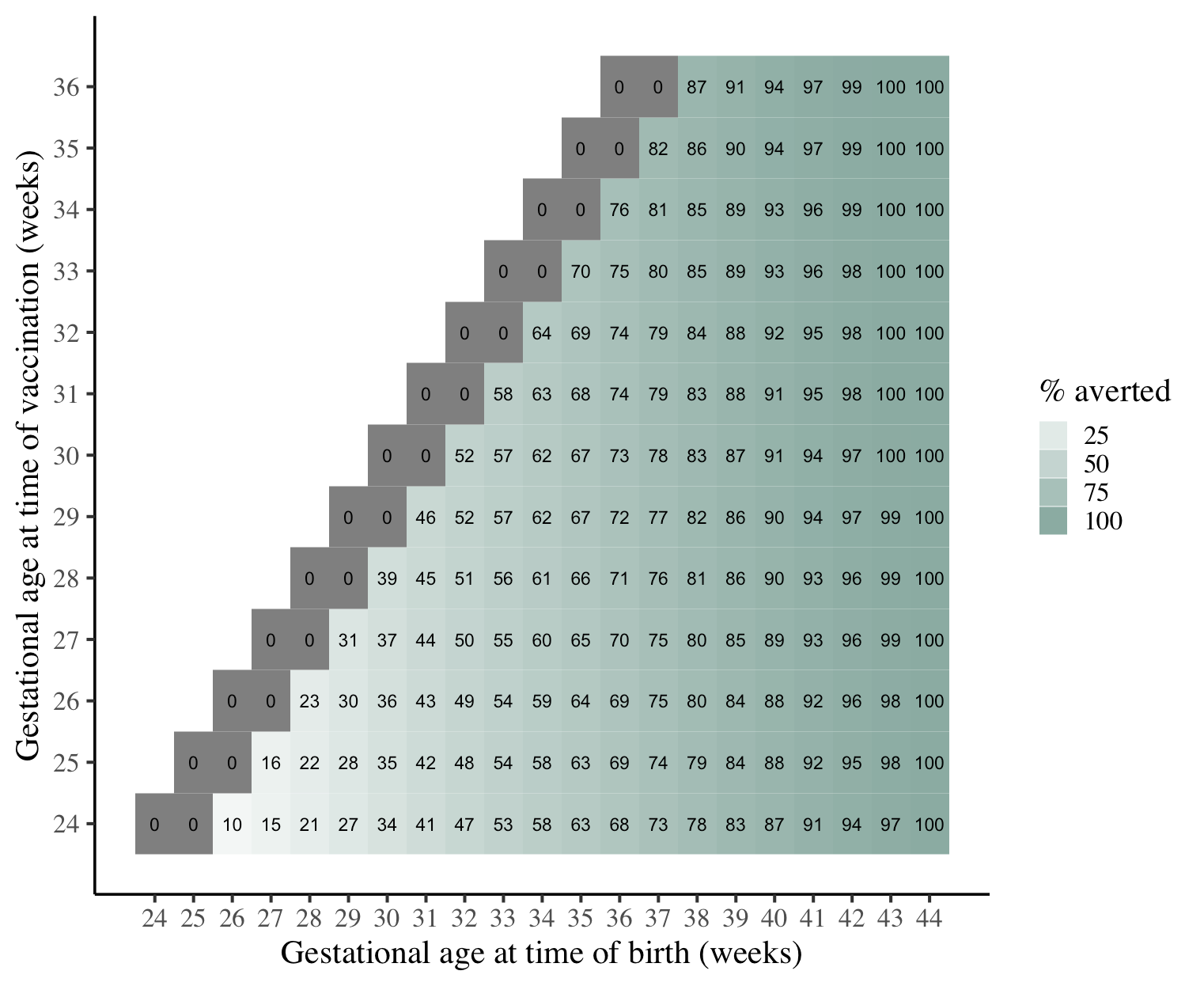
RSV-A
  2. RSV-B

Figure S1: The estimated percentage of in-hospital mortality cases averted by maternal vaccination for RSV-A (a) and RSV-B (b) in low-income and middle-income countries (LMICs) in the first six months of life, depending on timing of vaccination and gestational age at time of birth.


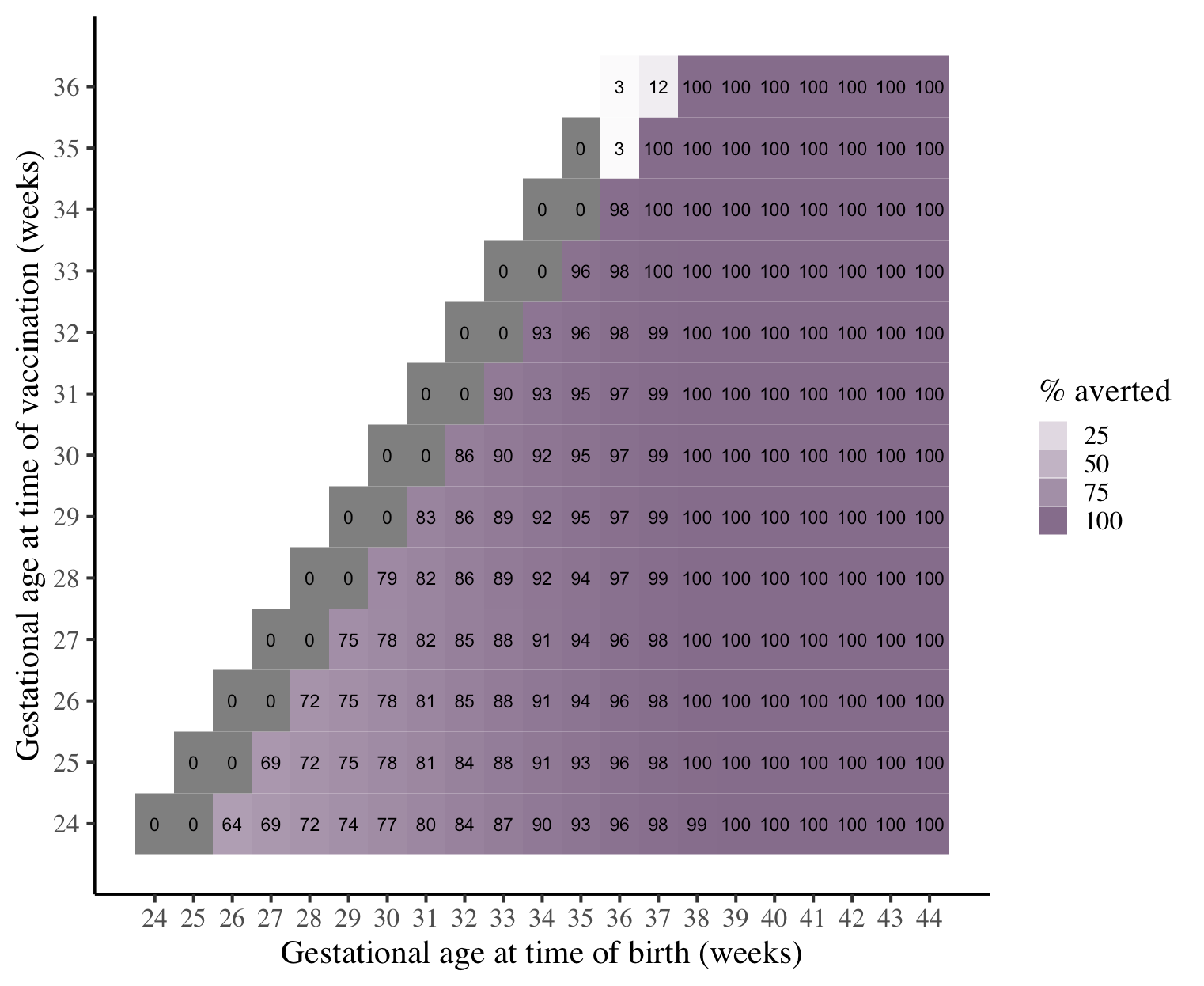


1.
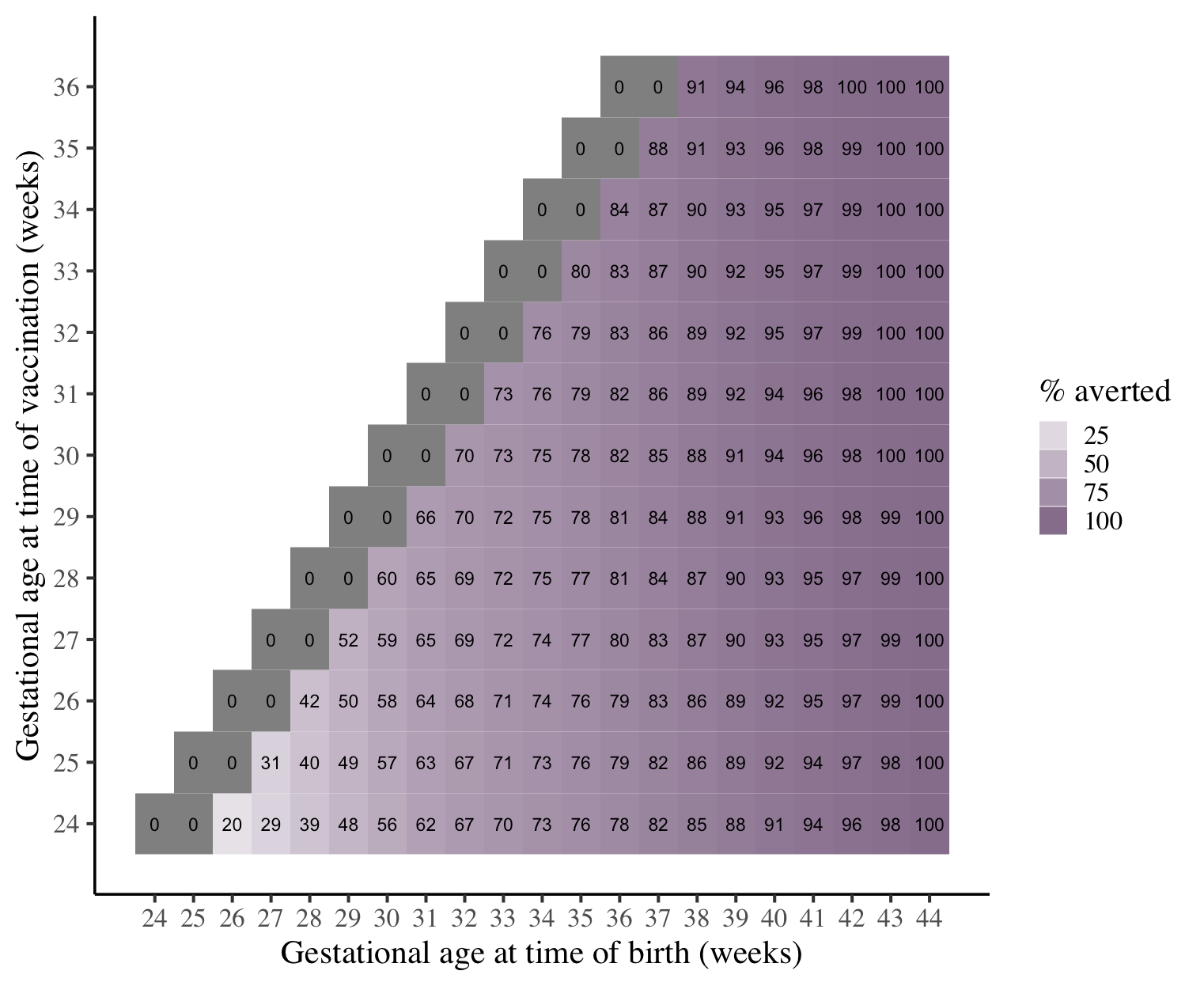
RSV-A
2. RSV-B

Figure S2: The estimated percentage of out-of-hospital mortality cases averted by maternal vaccination for RSV-A (a) and RSV-B (b) in LMIC in the first six months of life, depending on timing of vaccination and gestational age at time of birth.

*S4.2. RSV GOLD Database*


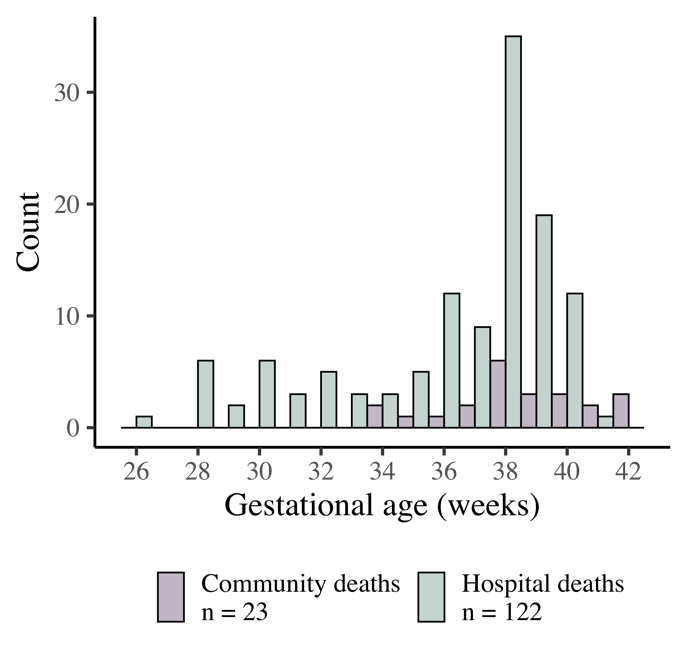

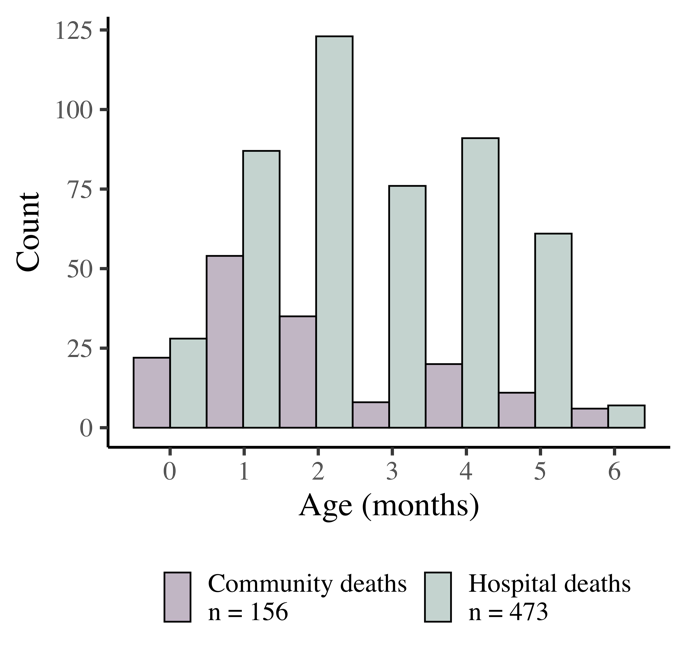


1. (b)

Figure S3: The frequency distribution of gestational age at time of birth (a) and the frequency distribution of age at time of death (b) in the RSV GOLD subset [4].


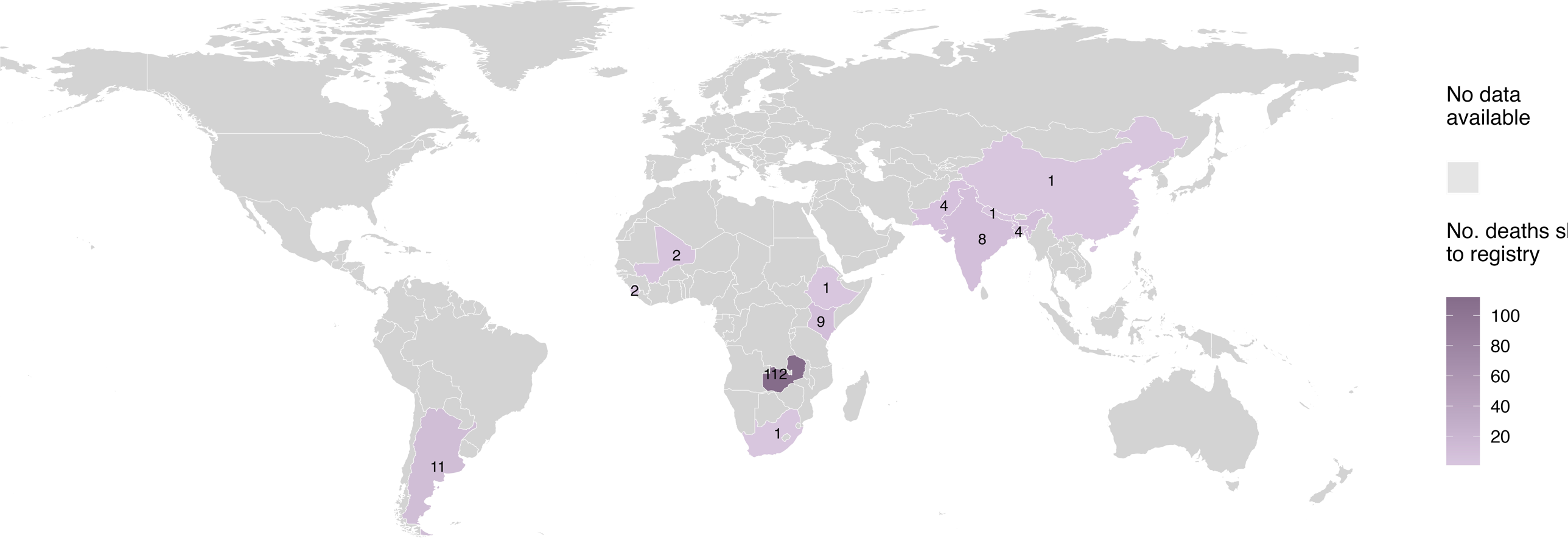


(a)


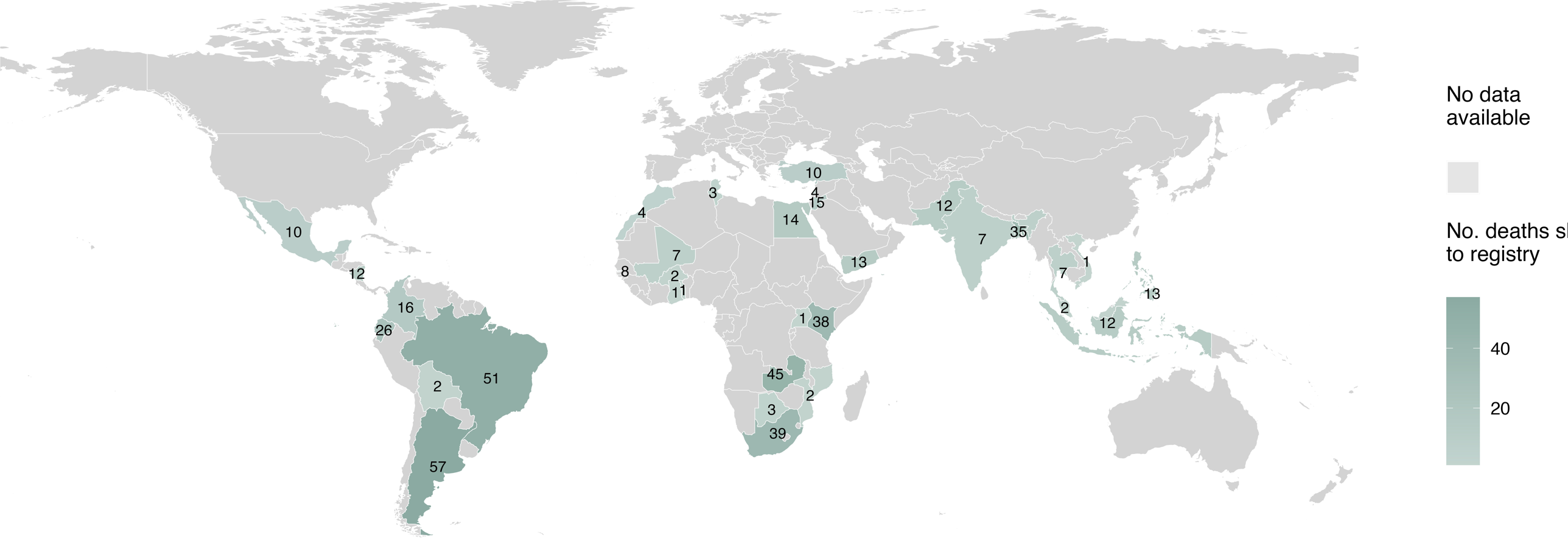


(b)

Figure S4: (a) World map showing L(M)ICs and UMICs that shared RSV-confirmed community deaths under 6 months of age and number of RSV-confirmed community deaths shared to the registry. The color gradient of purple indicates number of deaths shared, with darker purple representing increased number of deaths shared. Numbers of deaths are visible on the map. (b) World map showing L(M)ICs and UMICs that shared RSV-confirmed in-hospital deaths under 6 months of age and number of deaths of RSV-confirmed in-hospital deaths shared to the registry.

The color gradient of green indicates number of deaths shared, with dsa1rk9er green representing increased number of deaths shared. Numbers of deaths are visible on the map. Abbreviations: L(M)ICs, lower-income-lower-middle-income country; RSV, respiratory syncytial virus; UMIC,

upper-middle-income country.

*S4.3. Resampling age at time of death*


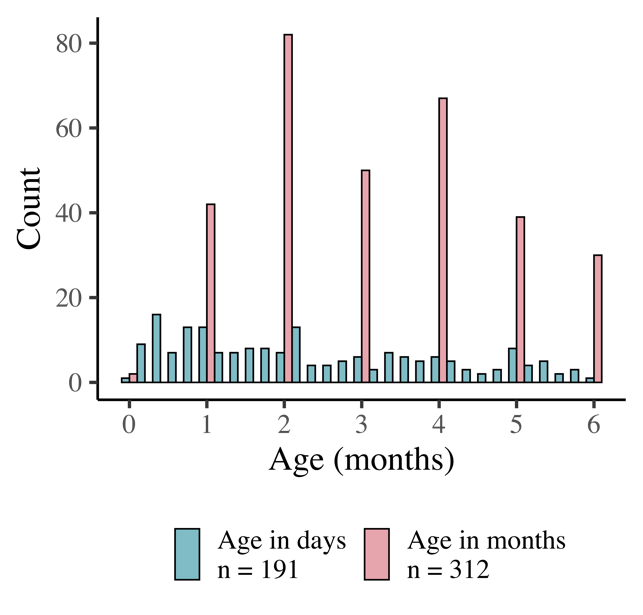

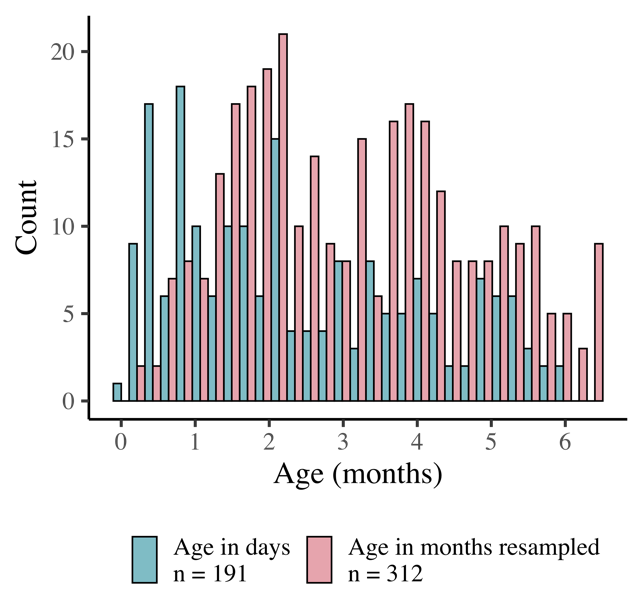


- 1.
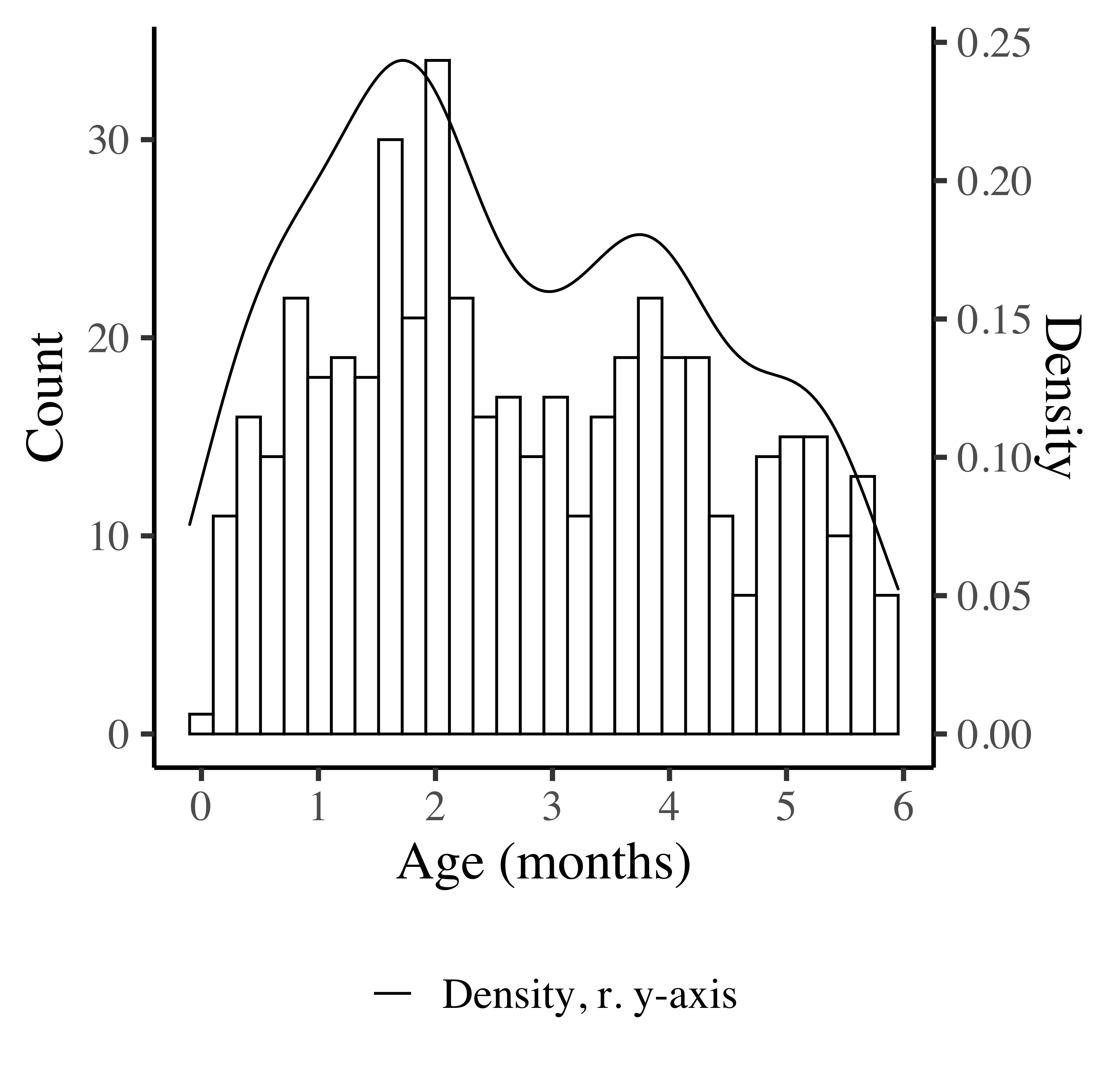
(b)

(c)

Figure S5: (a) Frequency distribution of the data reported in days (blue) and data reported in months (pink) in the RSV GOLD subset (b) Example of a dataset where the data reported in months (pink) is resampled (c) Frequency distribution of the partly resampled dataset and the subsequent estimated probability density function (r.axis)

*S4.4. Gestational age at time of birth distribution*


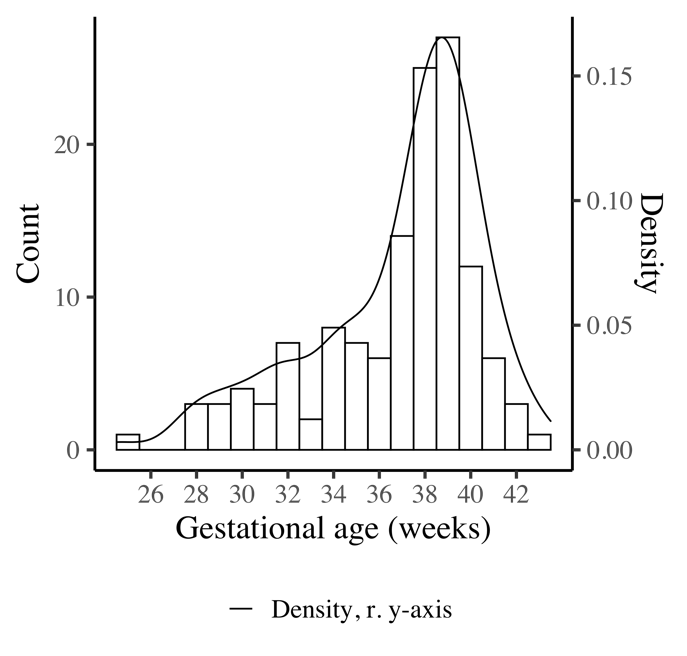

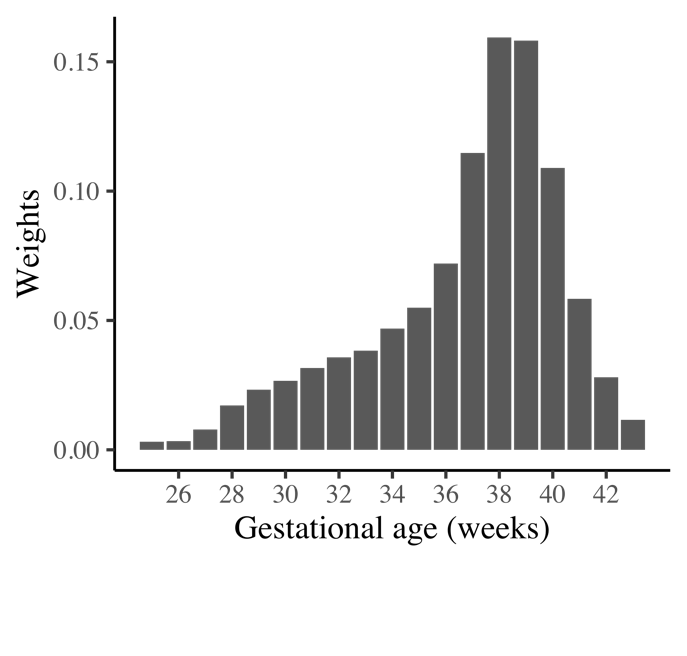


(a) (b)

Figure S6: (a) The frequency distribution of gestational age at time or birth for selected subset of most reliable observations in the RSV GOLD database and the subsequent estimated probability density function (r. y-axis) (b) The estimated probability mass function for gestational age.

**References**

[1] NaglerT,VatterT,NaglerMT.Package‘kde1d’2022;.

[2] Madhi S.A., Polack F.P., Piedra P.A., Munoz F.M., Trenholme A.A., Simões E.A., et al. Respiratory syncytial virus vaccination during pregnancy and effects in infants. N Engl J Med 2020;383(5):426–439.

[3] Li Y, Johnson EK, Shi T, Campbell H, Chaves SS, Commaille-Chapus C, et al. National burden estimates of hospitalisations for acute lower respiratory infections due to respiratory syncytial virus in young children in 2019 among 58 countries: a modelling study. The Lancet Respiratory Medicine 2021;9(2):175–85.

[4] Mazur N.I., Löwensteyn Y.N., Willemsen J.E., Gill C.J., Forman L., Mwananyanda L.M., et al. Global respiratory syncytial virus–related infant community deaths. Clin Infect Dis 2021;73(Suppl_3):S229–S237.
